# Short-duration selective decontamination of the digestive tract infection control does not contribute to increased antimicrobial resistance burden in a pilot cluster randomised trial (the ARCTIC Study)

**DOI:** 10.1136/gutjnl-2023-330851

**Published:** 2024-01-22

**Authors:** Iain Robert Louis Kean, John A Clark, Zhenguang Zhang, Esther Daubney, Deborah White, Paloma Ferrando-Vivas, Gema Milla, Brian Cuthbertson, John Pappachan, Nigel Klein, Paul Mouncey, Kathy Rowan, John Myburgh, Theodore Gouliouris, Stephen Baker, Julian Parkhill, Nazima Pathan, ARCTIC research team

**Affiliations:** 1 Department of Paediatrics, University of Cambridge, Cambridge, UK; 2 Cambridge University Hospitals NHS Foundation Trust, Cambridge, UK; 3 ICNARC, London, UK; 4 Sunnybrook Hospital, Toronto, Ontario, Canada; 5 University of Southampton, Southampton, UK; 6 University College London, London, UK; 7 The George Institute for Global Health, Newtown, New South Wales, Australia; 8 Department of Medicine, University of Cambridge, Cambridge, UK; 9 Department of Veterinary Medicine, University of Cambridge, Cambridge, UK; 10 ARCTIC research team, Cambridge, UK

**Keywords:** ANTIBIOTIC THERAPY, DRUG RESISTANCE

## Abstract

**Objective:**

Selective decontamination of the digestive tract (SDD) is a well-studied but hotly contested medical intervention of enhanced infection control. Here, we aim to characterise the changes to the microbiome and antimicrobial resistance (AMR) gene profiles in critically ill children treated with SDD-enhanced infection control compared with conventional infection control.

**Design:**

We conducted shotgun metagenomic microbiome and resistome analysis on serial oropharyngeal and faecal samples collected from critically ill, mechanically ventilated patients in a pilot multicentre cluster randomised trial of SDD. The microbiome and AMR profiles were compared for longitudinal and intergroup changes. Of consented patients, faecal microbiome baseline samples were obtained in 89 critically ill children. Additionally, samples collected during and after critical illness were collected in 17 children treated with SDD-enhanced infection control and 19 children who received standard care.

**Results:**

SDD affected the alpha and beta diversity of critically ill children to a greater degree than standard care. At cessation of treatment, the microbiome of SDD patients was dominated by Actinomycetota, specifically *Bifidobacterium,* at the end of mechanical ventilation. Altered gut microbiota was evident in a subset of SDD-treated children who returned late longitudinal samples compared with children receiving standard care. Clinically relevant AMR gene burden was unaffected by the administration of SDD-enhanced infection control compared with standard care. SDD did not affect the composition of the oral microbiome compared with standard treatment.

**Conclusion:**

Short interventions of SDD caused a shift in the microbiome but not of the AMR gene pool in critically ill children at the end mechanical ventilation, compared with standard antimicrobial therapy.

**WHAT IS ALREADY KNOWN ON THIS TOPIC**

- Selective decontamination of the digestive tract (SDD)-enhanced infection control has a significant impact on the reduction of ventilator-associated pneumonia during hospitalisation for critical illness.
- SDD reduces morbidity and mortality in adult intensive care units.
- Large studies examining the changes in antimicrobial resistance attributable to SDD have relied on microbial culture or quantitative PCR.

**WHAT THIS STUDY ADDS**

- This study observed no significant change in clinically relevant antimicrobial resistance gene abundance when patients were treated with SDD-enhanced infection control compared with standard care.
- Changes were observed in the gut microbiome of children treated with SDD, with perturbation continuing up to 2–3 months post-treatment.
- This six-site cluster randomised trial is the largest study to examine longitudinal changes to antimicrobial resistance genes using shotgun metagenomic sequencing.

**HOW THIS STUDY MIGHT AFFECT RESEARCH, PRACTICE OR POLICY**

- This study prepares the groundwork for a larger multicentre trial which can examine longer exposures to SDD-enhanced infection control in children.
- This study suggests that microbiome recovery may be delayed after SDD treatment.

## Introduction

The reduction of healthcare-associated infections (HCAIs) and improved antimicrobial stewardship are important for patient outcomes and improving global health.

The majority of HCAIs are caused by a small subset of opportunistic pathogens, generally *Staphylococcus aureus, Candida albicans* and Gram-negative aerobic rods (GNARs).^1 2^ Ventilator-associated pneumonia (VAP) was previously reported as the second most common form of HCAI after bloodstream infection, with VAP affecting between 10% and 20% of children admitted to paediatric intensive care unit (PICU) with a 30-day mortality of 30%.^3^ However, recent studies indicate that VAP is overtaking bloodstream infections in PICU settings contributing to 56% of all PICU HCAIs, leading to lengthened hospitalisation.^4^

Microbiological diagnosis of VAP is suboptimal with a high proportion of culture-negative samples, but in many cases, intestinal organisms are identified.^5^ Selective digestive tract decontamination (SDD, also described as selective decontamination of the digestive tract) aims to reduce the ingress of intestinal microorganisms, including GNAR and *Candida* spp into the lower respiratory tract of mechanically ventilated patients and reduce the risk of VAP.

Multiple reviews of clinical trials of adult patients have concluded that a full protocol of oral paste and gastric suspension SDD combined with intravenous antimicrobials was effective in reducing VAP compared with standard care (SC).^6–8^ The recent SuDDICU trial^9^ reported that SDD treatment reduced the incidence of phenotypical antimicrobial resistance (AMR) from cultured organisms and new bacteraemia compared with conventional treatment, but did not significantly affect overall mortality in adult intensive care units (ICUs).

Paediatric trials of SDD have generally been small, single-unit trials.^10–14^ Three^10–12^ out of four trials reported a reduction of infections from Gram-negative bacteria in the SDD group, while Barret *et al* 13 observed no difference in infection rate. To address the paucity of SDD data available in a paediatric clinical setting, the Paediatric Intensive Care and Infection Control (PICnIC) pilot clinical trial^15 16^ examined the feasibility for a future definitive clinical trial to examine the safety and efficacy of SDD-enhanced infection control to prevent HCAIs in critically ill children requiring at least 48 hours of mechanical ventilation.

In this pilot study, we outlined the baseline changes of AMR in critically ill children who had received mechanical ventilation and antimicrobial therapy and leveraged the wider PICnIC Study by examining the temporal changes on the microbiome composition and antimicrobial gene carriage in faecal and oropharyngeal compartments in a subset of critically ill children receiving either standard or SDD-enhanced infection control.

Having previously reported on PICU-derived changes in paediatric microbiomes through 16S rRNA gene sequencing,^17 18^ this study is the first to examine the proportional composition of microbiota linked to the pool of AMR genes and the effect of SDD-enhanced infection control both acutely and during recovery in critically ill children.

To study the effects of both standard and SDD-enhanced infection control on oropharyngeal and intestinal microbiota and the temporal effects on AMR gene carriage, paired oropharyngeal and rectal swabs were taken from a subset of critically ill children enrolled to a National Institute for Health and Care Research-funded pilot trial of SDD (the PICnIC trial, IRAS 239234, ISRCTN40310490^19^).

## Materials and methods

An extended method section is available in the online supplemental materials and methods.

10.1136/gutjnl-2023-330851.supp2 Supplementary data

### Trial design

The protocol for the pilot SDD trial was previously reported by Brown *et al*,^15^ (ISRCTN40310490^19^) with further details available from Pathan *et al*.^16^ The study consisted of five periods: prestudy ecology (1 week), baseline phase (phase one) (8 weeks), pre-intervention ecology (1 week), intervention phase (phase two) (9 weeks) and post-study ecology (1 week). The intervention phase was divided into SC and SDD, with three hospitals randomly assigned to each arm. All patients were administered standard antimicrobial therapy as needed. SDD was administered in addition to SC at 6-hour intervals. The SDD treatment consisted of an oral paste consisting of tobramycin (2% weight per volume (w/v)), colistin (2% w/v) and nystatin (2% w/v), and a suspension containing tobramycin (10 mg/mL), colistin (8 mg/mL) and nystatin (2×10^5^ IU/mL). SC in UK PICUs is the administration of a third-generation cephalosporin upon arrival in the PICU unless otherwise indicated.

### Enrolment and consent

In order to establish the immediate impact of critical illness on the microbiome, faecal samples were taken at admission in a cohort of critically ill children requiring mechanical ventilation for suspected lower respiratory tract infection.^5^ A delayed consent model was used, and informed consent was obtained within 48 hours of sample collection.

To evaluate the impact of SC and SDD-enhanced infection control on the paediatric faecal microbiome during and after critical illness, paired oropharyngeal and faecal samples were collected from a subset of children enrolled to the PICnIC pilot trial following informed consent, within 6 hours of admission (prior to SDD treatment), peri-extubation when SDD treatment was completed and in convalescence (6–8 weeks post-discharge).

Age-matched healthy control microbiomes were selected from previously published data from the USA (n=63).^20 21^

### Patient cohort

The PICnIC Study^16^ enrolled 368 children. Phase one recruited 207 children to SC, with phase two recruiting 161 children. Children recruited during phase two were divided by intervention with 94 recruited at SC units and 67 recruited at units administering SDD. This substudy enrolled 36 children across both trial phases. Phase one recruitment comprised three children to whom SC was administered, with the remaining 16 children recruited during phase two. All 17 SDD-treated patients were recruited during phase two.

### Sample collection

Oropharyngeal and rectal swabs were taken as soon as clinically feasible using a sterile cotton swab (MWE Medical Wire, Corsham, UK) and stored in sterile containers containing 1 mL of DNA/RNA Shield (Zymo Research, Irvine, California, USA). Oral swabs were used in place of oropharyngeal swabs for home collection kits. In children enrolled to the intervention arm of the PICnIC trial, pretreatment oropharyngeal swabs were performed after the initiation of mechanical ventilation but prior to first application of the oropharyngeal paste. Pretreatment rectal swabs from SDD patients were taken as soon as possible but within 6 hours of SDD treatment. Pretreatment swabs from SC patients were taken as soon as possible after mechanical ventilation. For all patients, when a clinical decision was made to end ventilation, treatment swabs were collected peri-extubation; oropharyngeal and rectal swabs were taken as soon as clinically possible once extubation was decided. Collected swabs were stored in 1 mL of DNA/RNA Shield. Where facilities permitted, swabs were stored at −80°C as soon as possible and shipped on dry ice, otherwise swabs were returned to Cambridge University Hospital at ambient temperature via priority mail. Post-PICU recovery samples were collected between 2 and 3 months after PICU discharge by families and returned directly to the University of Cambridge. Oral swabs were stored in 1 mL of DNA/RNA Shield, and faecal samples were collected using OmniGene Gut tubes (DNA Genotek, Ottawa, Ontario, Canada). Samples were returned to Cambridge University Hospital at ambient temperature by priority mail. Samples were received at the University of Cambridge and stored at −80°C until extraction.

### Sample processing and sequencing library preparation

Samples were extracted using the PowerSoil Pro DNA extraction kit (Qiagen, Hilden, Germany). For oropharyngeal, oral and rectal swabs, 400 µL of DNA/RNA Shield preservation medium-containing sample was extracted with 400 µL of extraction buffer. For OmniGene Gut tubes, 250 µL of medium-containing sample was extracted with 550 µL of extraction buffer.

DNA quantification was performed using a Qubit4 (ThermoFisher, Waltham, Massachusetts, USA) high-sensitivity double-stranded DNA assay. Sequencing libraries were prepared using the NEBNext Ultra II DNA library preparation kit (NEB, Ipswich, Massachusetts, USA) following the standard protocol and sequenced with a NovaSeq 6000 (Illumina, San Diego, California, USA).

### Data processing

Sequences were quality trimmed using trim_galore V.0.5.0,^22^ and host sequences were removed using Bowtie2 V.2.3.5.1^23^ and the GRCh38.p14 human reference genome.^24^ Metagenomic composition was determined from cleaned reads using Kraken2 V.2.0.9-beta^25^ and the nt database (05/05/2023). AMR genes were identified from paired-end reads using ARIBA V.2.14.6 and the CARD database V.3.2.7.^26 27^ When comparing intercohort datasets, we corrected for batch effect using Gibbons *et al*’’s percentile normalisation method.^28^

Statistical analysis was performed using R V.4.2.0.^29^ Read counts were normalised per million reads. Phylogenetic analysis consisting of non-metric dimensional scaling (nMDS) clustering of Bray-Curtis distances and alpha diversity was performed using vegan V.2.6-2.^30^ Clustering analysis was performed with the Adonis2 function, controlling for multiple measures where appropriate.^31^ Multiple comparisons of non-parametric variables were performed using repeated Wilcoxon tests with the paired test used as required for paired data. Multiple comparison adjustment was performed using the Benjamini-Hochberg method,^32^ and probability is reported as the q value (adjusted p value) in cases of multiple comparisons.^33^ Data transformation and plotting were performed using the tidyverse suite.^34^ MaAsLin2 V.1.10.0 was used for multivariate analysis.^35^ We chose to set alpha at a rate of 1 in 20 for p and q values.

### Analysis of resistance genes

To focus the analysis of AMR, genes involved in resistance to non-clinically relevant compounds, such as heavy metals, were excluded. Tetracyclines are not indicated for use in children due to the effects on bones and teeth.

Genes detected with ARIBA were compiled for each patient and measured in reads per kilobase_gene_ per megabase of sequencing (RPKM).

### Trial reporting

This report used the Strengthening the Reporting of Observational Studies in Epidemiology cohort reporting guidelines.^36^

An extended materials and methods section is available in the online supplemental materials and methods.

## Results

### Patient information

A baseline PICU population that comprised of 86 children admitted to the PICU between 2020 and 2022, for whom faecal samples were collected within the first 48 hours of PICU admission was examined for faecal microbiome composition and AMR gene carriage.^5^ Further patient demographics were reported by Clark *et al*.^5^ Samples from these patients were compared with the microbiomes of 63 age-matched healthy children^20^ (table 1).

**Table 1:** Patient information

Characteristic	Healthy controls (n=63)	PICU Baseline (n=86)	Standard care (n=19)	SDD (n=17)
Age (years), med (IQR)	1.2 (0.4–4.3)	1.2 (0.4–5.2)	0.2 (0.1–1.4)	0.5 (0.2–1.3)
Sex (male)	40 (63.5%)	56 (65.1%)	11 (57.9%)	11 (64.7%)
Weight (kg), med (IQR)	NR	10.0 (5.9–18.0)	5.0 (3.0–12.5)	6.0 (4.0–11.0)
Hospital stay				
PIM3 score,^62^ med (IQR)	–	3.2 (0.5–4.8)	3.2 (0.8–5.1)	3.2 (1.3–5.8)
Ventilation time (hours), med (IQR)	–	104.9 (67.0–188.6)	87.0 (60.5–125.0)	95.0 (73-157)
Ventilation-free hours*, med (IQR)	–	615.1 (531.7–653.0)	633.0 (595–659.5)	625.0 (563.0–647.0)
PICU-free days*, med (IQR)	–	NR	25.0 (22.5–26.0)	24.0 (20.0–25.0)
Healthcare-associated infection	–	13 (15.1%)	1 (5.3%)	1 (5.9%)
Nasogastric feed (at admission)	–	NR	6 (31.5%)	6 (35.3%)
Survival				
Hospital discharge (%)	–	84 (97.7)	19 (100)	17 (100)
30 days post-PICU (%)	–	NR	19 (100)	17 (100)
Primary admission				
Cardiovascular	–	5	3	0
GI	–	1	1	0
Haematological/oncological	–	3	0	2
Infection	–	7	5	3
Neurological	–	9	1	0
Postoperative	–	5	0	0
Respiratory	–	53	8	11
Trauma	–	3	0	0

Data presented as median values with 25th and 75th centile.

*Days free from PICU or ventilation at 30 days post-admission.

NR, not recorded; PICU, paediatric intensive care unit; PIM3, Paediatric Index of Mortality 3; SDD, selective decontamination of the digestive tract.

**Figure 1:**
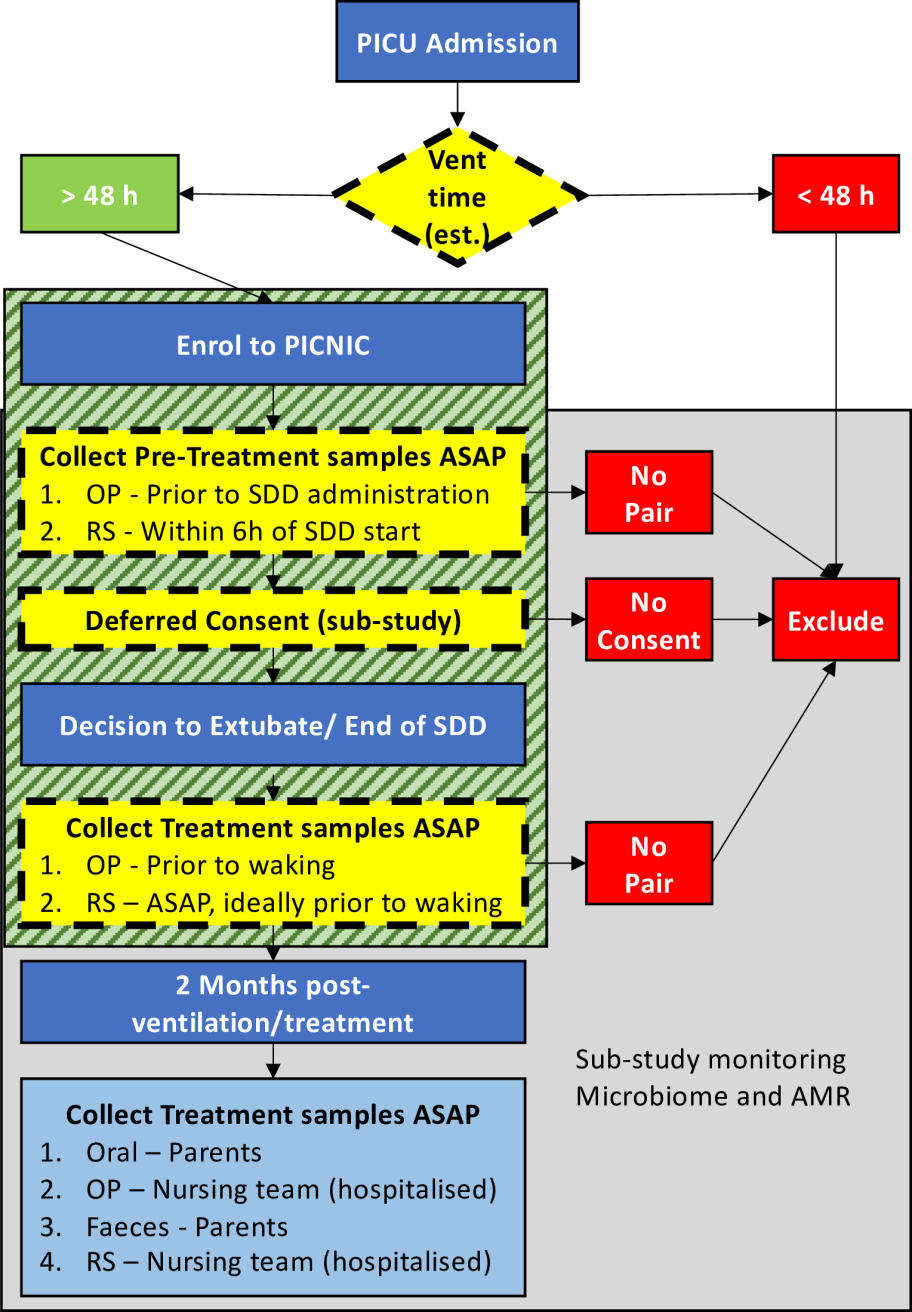
PICnIC AMR and microbiome monitoring substudy enrolment schema. Children became eligible for the substudy upon enrolment to the PICnIC Study. Inclusion into PICnIC is outlined by Brown *et al* 15 and Pathan *et al*.^16^ Briefly, children arriving in the PICU who required mechanical ventilation were assessed by clinicians, and patients judged to require greater than 48 hours of invasive respiratory support became eligible. Patients were not eligible if they were known to be allergic to colistin, tobramycin or nystatin, or if they were known to be colonised by a microorganism resistant to any of the three previous drugs. Once enrolled to PICnIC, children became eligible for this substudy. This substudy used a deferred consent model for collecting research samples. Research nurses were instructed to collect pretreatment samples as soon as clinically feasible. Oropharyngeal samples were to be taken before the application of SDD paste in the case of treatment patients and as soon as possible for standard care patients. Rectal swabs were to be taken as soon as possible but within 6 hours of admission for all patients. Six hours was chosen as the transit time of the non-absorbable SDD drugs from the stomach to rectum should not have occurred within this window. Parents/guardians were approached for consent as soon as was reasonable. Samples collected from children with no deferred consent were destroyed. Patients who were extubated and stopped SDD prior to consent being collected were excluded from the trial. If consent was received, treatment samples were advised to be collected as soon as a clinical decision was made to end mechanical ventilation. This time point was chosen as collection of samples from sedated children would impart the least amount of discomfort. The substudy required that both pairs of samples be collected. Any child who was missing one or more of the first pairs of samples was excluded from the study. Recovery samples were scheduled for 2–3 months post-SDD cessation. For recovery samples, parents were asked to collect an oral swab in place of the oropharyngeal swab, because it can be easily performed by non-specialists on small children. Parents were also asked to collect a faecal sample using the OMNIGene Gut tube from DNAGenotek. For children who continued to stay in the PICU after extubation, nurses collected OP and RS, unless faeces were passed. Collection of recovery samples was not an exclusion criterion. Boxes with dashed outlines indicate inclusion/exclusion points. The box with diagonal lines indicates the minimum requirements for inclusion to this substudy. AMR, antimicrobial resistance; ASAP, as soon as possible; OP, oropharyngeal; PICnIC, Paediatric Intensive Care and Infection Control; PICU, paediatric intensive care unit; RS, rectal swab; SDD, selective decontamination of the digestive tract.

The study population comparing SDD with SC was formed of 36 children from whom serial samples were collected (figure 1). Of these, 17 received SDD and 19 received SC. Primary admission diagnoses are shown in table 1. Antimicrobial use is summarised in online supplemental figure 1A. The PICnIC trial was a pilot trial to assess the feasibility of administering SDD on a unit-wide basis in PICUs in England and was not powered for clinical significance. This substudy was also a pilot project to identify the feasibility of monitoring the microbiome and AMR of critically ill children receiving SDD-enhanced infection control. The strict criteria required for serial sample collection reduced our enrolment to 36 patients (figure 1).

10.1136/gutjnl-2023-330851.supp1 Supplementary data

### Faecal microbiome composition but not AMR gene carriage at PICU admission is impacted by antimicrobial administration

**Figure 2:**
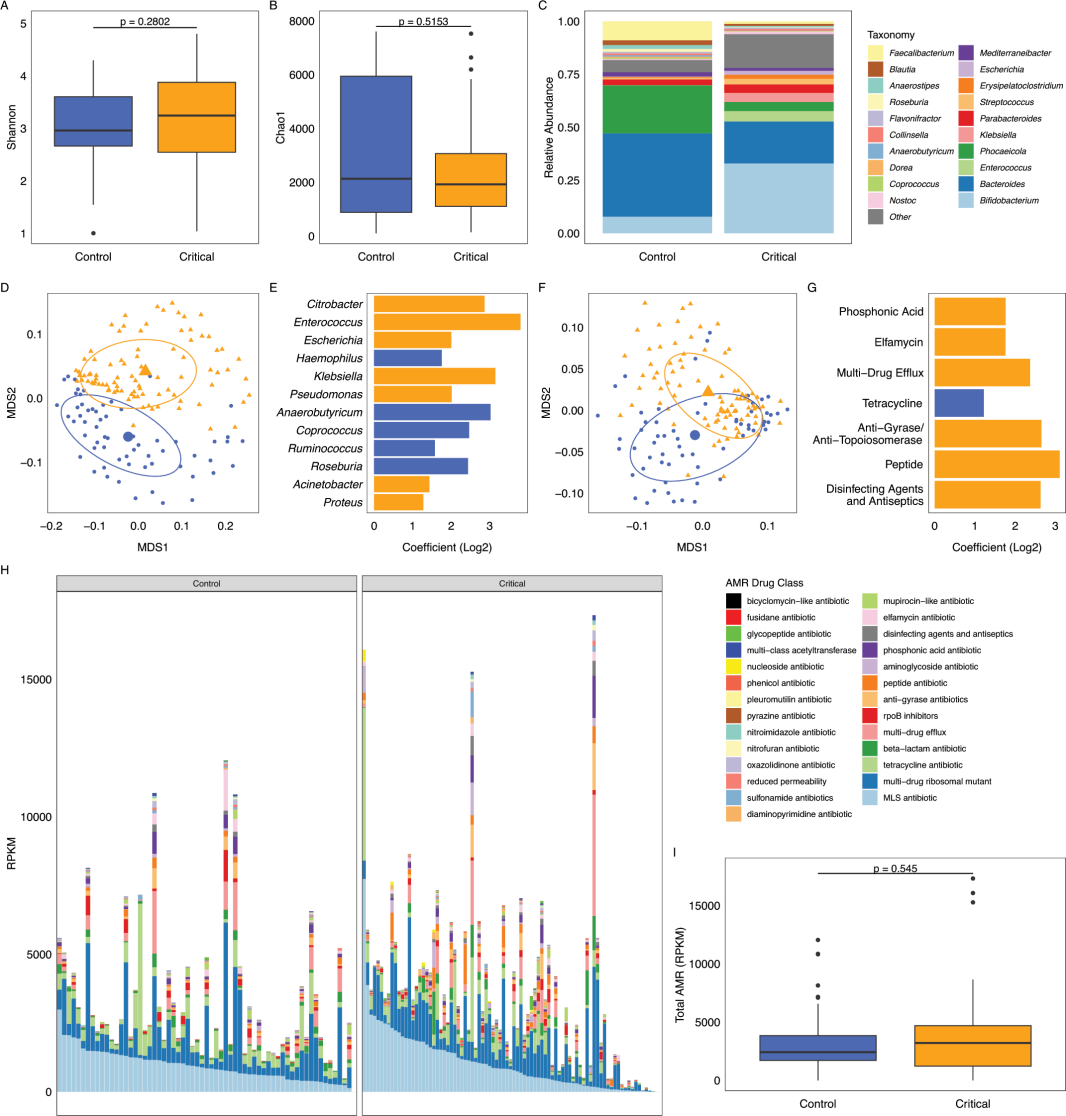
Microbiome diversity and AMR gene pool of critically ill children. Critically ill children admitted to the Cambridge University Hospital paediatric intensive care unit were sampled to identify the composition of their microbiome and resistome. Healthy control children were selected from the NIH RESONANCE trial conducted in the USA. (A) Microbiome alpha diversity (Shannon’s Index) is comparable between admission samples of critically ill children and healthy control children. Sequences were classified using Kraken2 with a confidence level of 0.1 and the 05/2023 nt library. (B) Chao1 index of alpha diversity. (C) The microbial composition of critically ill children is divergent from age-matched healthy control children. Critically ill children have increased proportions of opportunistic pathogens and lower proportions of commensal Gram-negative anaerobes. We selected the top 10 most abundant bacteria from healthy controls and the top 10 most abundant bacteria in critically ill children not represented in list one, grouping all other microbiota into ‘other’. The median proportion of each genus is represented in the stacked bar chart. (D) Beta diversity indicates that the composition of both groups of children are divergent, with a statistically separate centroid clustering (PERMANOVA p=0.001, ellipse level set to 60%). Orange triangles represent critically ill patients, and blue circles healthy control samples. (E) MaAsLin2 differential representation of selected opportunistic pathogens responsible for VAP, representative secondary fermenters of the lower GI tract and significantly different intrinsically colistin-resistant bacteria. (F) nMDS plotting of Bray-Curtis distances of AMR genes measured as reads per kilobase of gene per megabase of sequencing (RPKM) and identified by ARIBA analysis. (G) Differently enriched AMR gene classes identified by MaAsLin2 between healthy controls and critically ill children. (H) AMR gene burden measured as RPKM for AMR compound structural classes and generalised function. Genes were pooled by target antimicrobial drug class. (I) Total RPKM between groups. AMR, antimicrobial resistance; MLS, macrolide, lincosamide and streptogramin B; NIH, National Institutes of Health; nMDS, non-metric dimensional scaling; PERMANOVA, permutational analysis of variance; VAP, ventilator-associated pneumonia.

Despite the high level of antimicrobials administered to critically ill children, alpha diversity (Shannon’s Index) in admission samples remained comparable with healthy controls (figure 2A). This trend was also observed with Chao1 (figure 2B). Critically ill children had increased representation of opportunistic pathogens such as *Enterococcus*, *Klebsiella* and *Escherichia*, and reduced abundance of complex carbohydrate fermenters such as *Bacteroides*, *Phocaeicola, Faecalibacterium* and members of the *Lachnospiraceae* (figure 2C). When comparing beta diversity calculated as Bray-Curtis distance using nMDS plotting, we observed distinct separation of study groups after applying the correction by Gibbons *et al* 28 to correct for technical factors (permutational analysis of variance (PERMANOVA) p=0.002; figure 2D). MaAsLin2 analysis indicated that many opportunistic pathogens including those with intrinsic resistance to colistin were significantly enriched in our baseline critically ill children, and secondary fermenters were decreased (figure 2E, all values of q<0.05).

When comparing the AMR profiles of US healthy control children and UK critically ill children, we observe difference in their composition with significant separation of AMR profiles based on gene family (figure 2F; PERMANOVA p<0.001). When comparing the class of AMR target with MaAsLin2, we observed that the gut microbiota of critically ill children had increased levels of resistance genes to drugs in the classes phosphonic acids, elfamycins, peptide antimicrobials, gyrase and topoisomerase inhibitors, and disinfecting agents along with multidrug efflux pumps. The control microbiomes were enriched for genes conferring resistance to tetracyclines (figure 2G, all q values of <0.05). We observed no significant difference in the median total AMR gene burden (RPKM) between antimicrobial-exposed admission samples of critically ill children and age-matched healthy children (p=0.545; figure 2I). From these results, we endeavoured to understand the changes manifested by the administration of standard antimicrobial therapy and SDD-enhanced infection control longitudinally on the microbiome and resistome.

### SDD is associated with distinct compositional alterations in the lower GI microbiome

**Figure 3:**
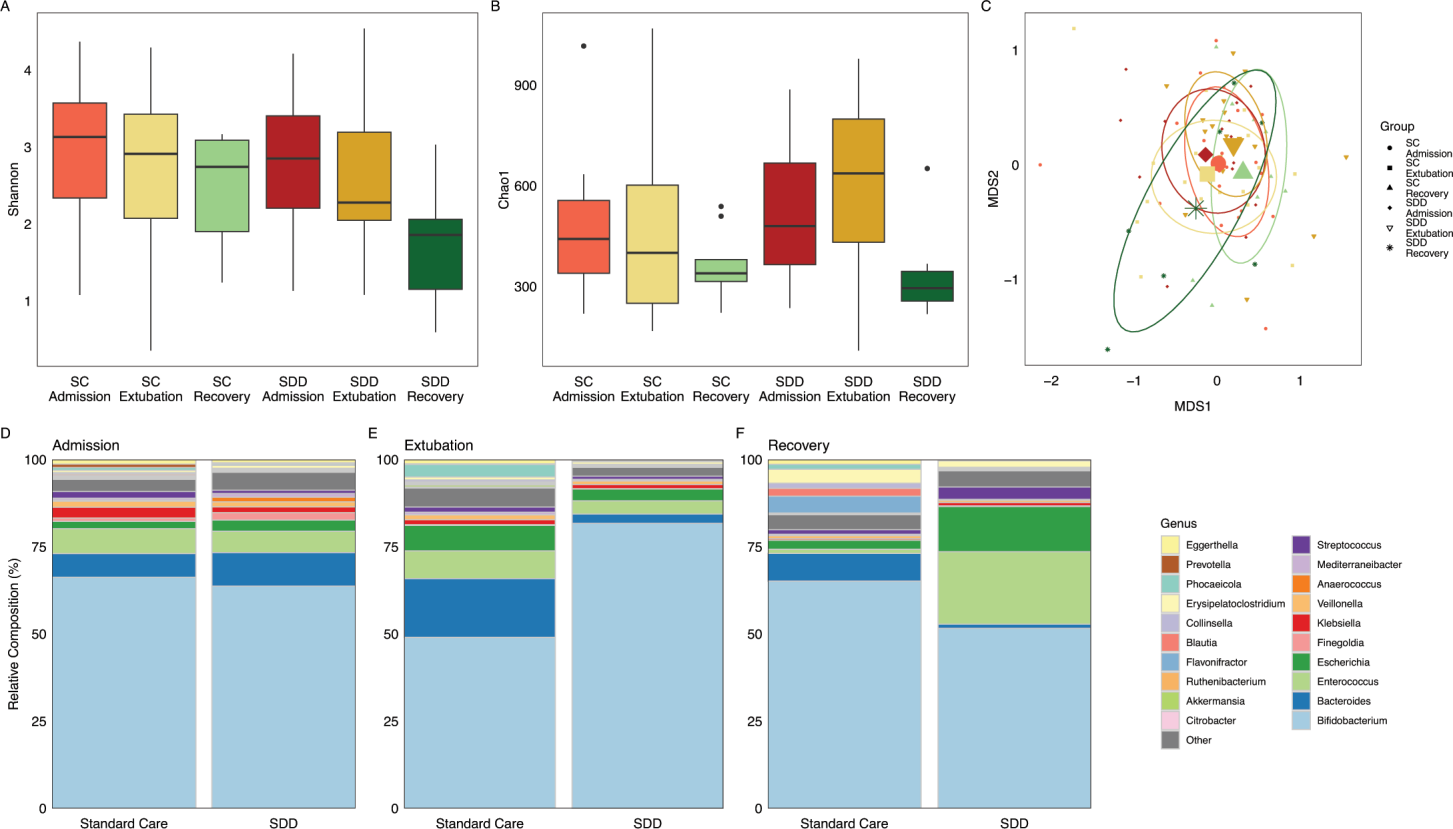
Changes in the lower GI microbiome during SDD-enhanced infection control in critically ill children. (A) Alpha diversity of patients calculated at species level using Shannon’s Index. No significant difference was observed after correcting for multiple comparisons. (B) Chao1 index of microbial richness. No significant difference was observed after correcting for multiple comparisons. (C) nMDS plot of beta diversity clustering of faecal microbiomes based on Bray-Curtis distances. No significant difference was observed in the clustering of any group after multiple corrections. Diamond=SDD admission, inverted triangle=SDD extubation, asterisk=SDD recovery, circle=SC admission, square=SC extubation, triangle=SC recovery. (D) Composition of the lower GI microbiome of patients at admission compared with healthy controls. The 10 most abundant families of microbiota by median proportion in the GI microbiome of standard care admission patients, and the 10 most abundant microbiota families in SDD admission patients by median proportion not represented in the first list were selected with the remaining taxa combined as ‘other’. (E) Lower GI composition of patients at extubation. Taxa are identified as in D. (F) Microbiome composition as median proportions at recovery. Taxa identified as in D. nMDS, non-metric dimensional scaling; SC, standard care; SDD, selective decontamination of the digestive tract.

No statistically meaningful fluctuation was observed in the alpha diversity of children receiving SC measured by Shannon’s Index (figure 3A). In contrast, the median alpha diversity of SDD patients declined over treatment and remained lower at 2–3 months post-treatment than controls (figure 3A). No significant difference was observed for any alpha diversity index or pairwise comparison therein after false discovery rate (FDR) correction (figure 3A,B). The beta diversity of SC patients diverges between admission and extubation samples, but the recovery samples cluster tightly with the admission samples (figure 3B). The beta diversity of children receiving SDD-enhanced infection control clustered closely at admission and extubation; however, there was a large divergence upon recovery from admission samples. After accounting for pairwise observations, no clusters were statistically separated by multiway PERMANOVA after FDR adjustment. Relevant beta diversity comparisons are illustrated in online supplemental figure 2.

Children who received SDD-enhanced infection control (n=17) had 10 dominant taxa whose median composition accounted for the majority of gut microbial composition (figure 3D–F). At the genus taxonomic level, these 10 taxa based on sequencing read abundance were: *Bifidobacterium, Bacteroides, Enterococcus, Escherichia, Finegoldia, Klebsiella, Veillonella, Anaerococcus*, *Mediterraneibacter* and *Streptococcus*. As a comparator of our clinical cohort, we selected the 10 taxa with the largest median abundance in the SC admission group not represented in the top 10 control bacteria: *Eggerthella, Prevotella, Phocaeicola, Erysyphiloclostridium, Collinsella, Blautia, Flavonifactor, Ruthenibacterium, Akkermansia* and *Citrobacter.* At admission, a high degree of similarity between the median profiles of SC patients and SDD patients was observed (figure 3C and online supplemental figure 3). Analysis of admission microbiomes using MaAsLin2 identified significantly increased *Acinetobacter* (q=7.356×10^−9^, coefficient=3.17), *Finegoldia* (q=5.579×10^−6^, coefficient=2.52), *Peptoniphilus* (q=3.696×10^−3^, coefficient=1.74) and *Rothia* (0.0045, coefficient=0.63) in the SDD cohort. At extubation, the median relative abundance of *Bifidobacterium* was highly divergent between treatments; however, none of the 20 most abundant taxa from admission samples were significantly different after correction for multiple tests (figure 3D and online supplemental figure 4). MaAsLin2 analysis of the extubation microbiome identified enrichment of *Dialister* (q=1.123×10^−5^, coefficient=2.54), *Lacticaseibacillus* (q=5.27×10^−5^, coefficient=2.62), *Sellimonas* (q=8.518×10^−5^, coefficient=2.22) and *Clostridium* (q=0.0373, coefficient=0.96) in SC patients, while SDD patients had increased *Stutzerimonas* (q=0.01811, coefficient=4.27)*, Acinetobacter* (q=0.0249, coefficient=4.61), *Paracoccus* (q=0.0249, coefficient=4.07) and *Ruthenibacterium* (q=0.0322, coefficient=0.99); however, median levels of *Acinetobacter, Stutzerimonas* and *Paracoccus* in both groups were below 0.01% by relative proportion. The compositional difference between SDD and SC patients at phylum, family, genus and species level can be observed in online supplemental figure 5.

At recovery, the SDD microbiomes were dominated by *Bifidobacterium* (figure 3F); however, this difference was not significant from SC (online supplemental figure 6). No significant difference in bacterial composition was identified in the top 20 bacteria (corrected for multiple comparisons). The changes in microbiome of patients receiving SDD-enhanced infection control who returned a recovery sample can be seen in online supplemental figure 7.

### SDD does not affect overall composition in the oropharyngeal microbiome

**Figure 4:**
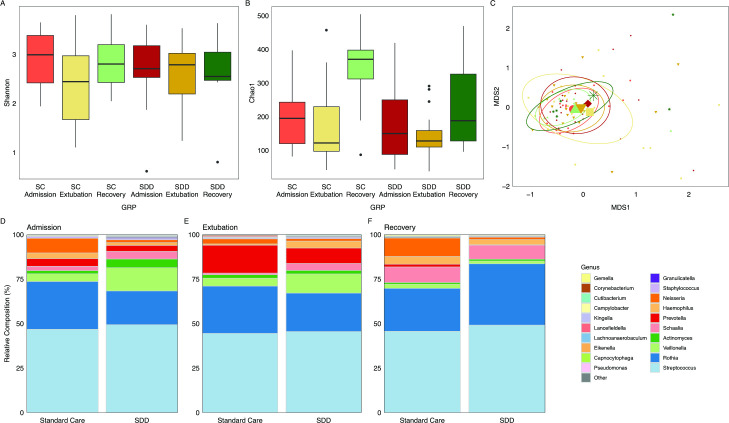
Changes in the oral microbiome during SDD-enhanced infection control in critically ill children. (A) Alpha diversity in SC patients measured as Shannon’s Index at species level at admission, extubation and recovery 2–3 months post-admission. (B) Chao1 index of microbial richness. (C) Microbiome beta diversity of PICU patients receiving SC. No statistical difference was calculated by PERMANOVA. Diamond=SDD admission, inverted triangle=SDD extubation, asterisk=SDD recovery, circle=SC admission, square=SC extubation, triangle=SC recovery. (D) Composition of the oral microbiome of patients at admission compared with healthy controls. The 10 most abundant families of microbiota by median proportion in the oropharynx of SDD patients at admission, and the 10 most abundant microbiota families in SC admission patients by median proportion not represented in the first list were selected with the remaining taxa combined as ‘other’. (E) Oral microbiome composition of patients and controls at extubation. Taxa are identified as in D. (F) Microbiome composition as median proportions at recovery. Taxa identified as in D. MDS, metric dimensional scaling; PERMANOVA, permutational analysis of variance; PICU, paediatric intensive care unit; SC, standard care; SDD, selective decontamination of the digestive tract.

The oropharyngeal microbiome can be considered a low-biomass biome, as such the majority (80%, IQR 66–86%) of shotgun metagenomic sequencing reads were from host DNA. After correcting for host reads and normalisation, we examined the alpha and beta diversity of the oropharyngeal microbiome. No significant difference in alpha diversity was observed in either the SC or SDD group at all time points for Shannon’s Index or Chao1 (figure 4A,B). We observed very little deviation in the beta diversity of oropharyngeal microbiomes of patients in either treatment group or between sampling time points (figure 4C and online supplemental figure 8). In the SC group at admission, a dominance of *Streptococcus* and *Rothia* with high amounts of *Neisseria* was observed (figure 4D). In the SDD patients, the 10 most abundant taxa were *Streptococcus, Rothia, Veillonella, Actinomyces, Schaalia, Prevotella, Haemophilus, Neisseria, Staphylococcus* and *Granullicatella* (figure 4D). MaAsLin2 identified higher carriage rates of *Pseudomonas* in SDD patients (q=3.998×10^−5^, coefficient=2.15) and *Corynebacterium* in SC patients (q=1.259×10^−4^, coefficient=1.65). At extubation, SC patients had expanded representation of *Prevotella, Actinomyces* and *Corynebacterium.* This was combined with a reduction in the median proportion of *Neisseria, Haemophilus* and *Schaalia* (figure 4D). An expansion of *Streptococcus* and *Prevotella* was observed in the SDD group at extubation, paired with a reduction in the median proportion of *Actinomyces* and *Veillonella* (figure 4E). MaAsLin2 analysis of the oral microbiome indicated significantly increased *Gemella* (q=4.265×10^−10^, coefficient=2.99) and *Haemophilus* (q=5.20×10^−7^, coefficient=2.38) in SDD patients. The microbiome of SC children at recovery was similar to their admission microbiome (figure 4F). SDD oral microbiomes at recovery had increased median *Rothia* (figure 4F). No significant differences were observed for any of the top 20 oral bacterial families between SC and SDD patients at each time point (online supplemental figures 9–11). MaAsLin2 did not identify any bacterial taxa with significant difference between treatment groups at recovery.

### SDD does not increase GI AMR gene carriage burden compared with standard infection control in critically ill children

**Figure 5:**
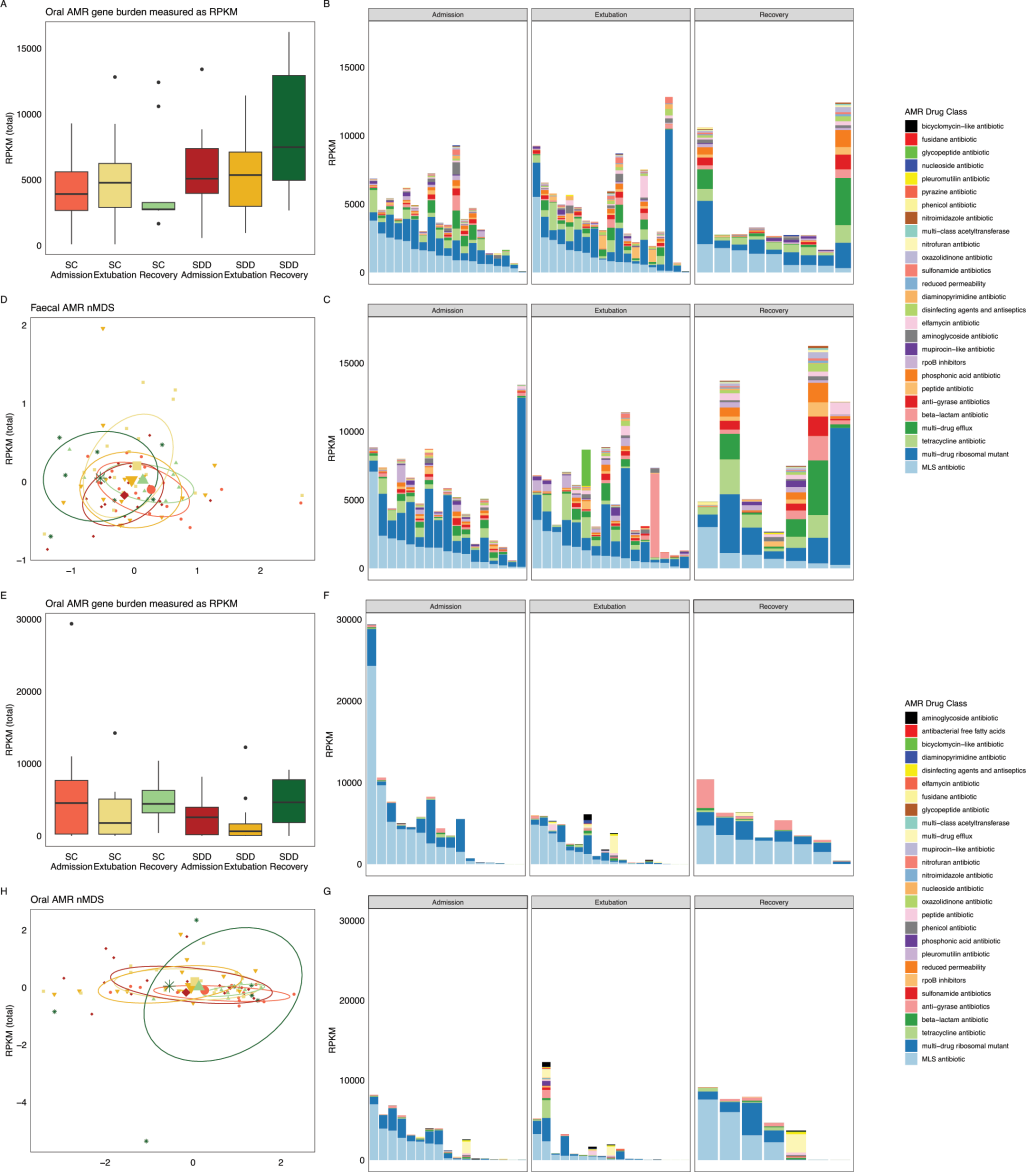
AMR gene proportion and composition from faecal and oral samples. AMR genes were assembled by ARIBA to the MEGARes V.3.0 library and normalised as reads per kilobase per megabase of sequencing (RPKM). (A) Total AMR gene burden in the lower GI microbiome. The sum of all AMR gene RPKM was compared across all groups. (B) RPKM of each AMR class detected in the faecal microbiome of individuals with SC. (C) RPKM of each AMR class detected in the faecal microbiome of individuals with SDD-enhanced infection control. (D) nMDS of AMR genes in faecal samples identified by ARIBA using the CARD V.3.2.7 database. Diamond=SDD admission, inverted triangle=SDD extubation, asterisk=SDD recovery, circle=SC admission, square=SC extubation, triangle=SC recovery. (E) Total AMR gene burden in the oropharyngeal microbiome. The sum of all AMR gene RPKM was compared across all groups. (F) RPKM of each AMR class detected in the oropharyngeal microbiome of individuals with SC. (G) RPKM of each AMR class detected in the oropharyngeal microbiome of individuals with SDD-enhanced infection control. (H) nMDS of AMR genes in oral samples identified by ARIBA using the CARD V.3.2.7 database. Diamond=SDD admission, inverted triangle=SDD extubation, asterisk=SDD recovery, circle=SC admission, square=SC extubation, triangle=SC recovery. AMR, antimicrobial resistance; MLS, macrolide, lincosamide and streptogramin B; nMDS, non-metric dimensional scaling; SC, standard care; SDD, selective decontamination of the digestive tract.

We found no significant difference in the median total RPKM across time points and treatment groups (figure 5). Resistance genes against 25 different drug classes and pathways of multidrug resistance were identified in our study (figure 5). The major component of most AMR profiles was macrolide–lincosamide–streptogramin (MLS) resistance (figure 5B and online supplemental figure 14). The second most abundant resistance class was ribosomal subunits with mutations conferring resistance against two or more drug classes followed by non-ribosomal tetracycline resistance, multidrug efflux pumps, beta-lactam and peptide antibiotic resistance (figure 5, online supplemental figure 12B,C and online supplemental figure 14).

No *mcr* colistin resistance genes were detectable in the microbiome of our patients in this study and we observed no major change in the proportion of colistin-resistant organisms across treatment groups or time points.

At admission, MaAsLin2 identified significantly more representation of membrane porins with reduced permeability to beta-lactams (q=8.76×10^−4^, coefficient=1.31), triclosan-resistant *gyrA* (q=8.76×10^−4^, coefficient=1.30) and daptomycin-resistant *gshF* (q=9.51×10^−4^, coefficient=1.21). From extubation samples, MaAsLin2 identified significantly increased representation of tetracycline protection proteins (*tetO, tetM, tetW, tet32*) (q=1.06×10^−5^, coefficient=1.63) and *ctx-M* beta-lactamases (q=0.0455, coefficient=1.01) in SC patients, while SDD patients had an increase in fluroquinolon-resistant *parC* (q=3.295×10^−5^, coefficient=1.67). No significant differences in AMR genes between treatment groups were observed at recovery. The majority of AMR genes classified as resistant to peptide antimicrobials were identified as daptomycin or lysocin resistance genes in Gram-positive organisms. We identified the gene families *arnA*, *arnT*, *basR*, *basS*, *cprR*, *cprS*, *eptA*, *eptB*, *pmrF* and *ugd* which are involved in 4-amino-4-deoxy-L-arabinose biosynthesis and polymyxin resistance in the faecal microbiomes of our patients. No genes were significantly different between groups after correcting for multiple comparisons (online supplemental figure 15). Of the 35 beta-lactam resistance genes identified, 23 were beta-lactamases. Examining the gene families *acc, act, ampC, blaZ, cdd, cmy, ctx-M, cblA, cepA, cfiA, cfxA, dha, ec, len, mal, ndm, orn, oxa oxy, pdc, pla, shv* and *tem*, we observed no significant difference between gene family presence after correction for multiple comparisons (online supplemental figure 16). The aminoglycoside resistance genes identified from sequencing *were **aac(3)-IId**, **aac(6’)-Ib7**, **aac(6’)-If**, **aac(6’)-Ii**, **aac(6’)-Im**, **aac(6)-Ie-aph(2)-Ia**, **aac(6’)-IB-Su, ant(2’’)-Ia**, ant(3’’)-Iia, ant(4’)-Ia, ant(6)-Ia, ant(6)-Ib, ant(9)-Ia, **aph(2’’)-Iia, aph(2’’)-If, aph(2’’)-Ig**, aph(3’’)-Ib, aph(3’)-IIIa, aph(3’)-Iia, aph(3’)-Iib, aph(3’)-Ia, aph(6)-Id, crcB*, *Paer-emrE, aadA, aadA13, aadA2, aadA24, aadA5, aadS, acrD, kdpD* and *kdpE* (genes conferring resistance to tobramycin highlighted in bold text). No significance was observed after corrections for multiple comparisons (online supplemental figure 17).

### SDD does not impact AMR gene carriage burden in oropharyngeal microbiome compared with SC in critically ill children

The abundance of AMR in the oropharyngeal microbiome is comparable, when normalised to RPKM than in the gut microbiome (online supplemental figure 12D). The burden of AMR at admission for SC and SDD patients was similar, and both treatment groups were observed to have reduced AMR burden at the end of treatment with SDD having a higher RPKM than SC. In recovery samples, we can see increased RPKM in both groups, but not a significant difference. The most common clinically relevant resistance detected in the oropharynx was MLS resistance, followed by multicompound resistant ribosomal mutants, tetracyclines and beta-lactams (online supplemental figure 12F,G). MaAsLin2 identified no significant differences between groups for AMR gene classes at admission and extubation. At recovery, *mfs* export pumps (q=6.068×10^−6^, coefficient=4.25), tetracycline ribosomal protection proteins (q=5.285×10^−5^, coefficient=2.84) and *msr* transporters (q=0.0289, coefficient=6.58) were significantly increased in SDD patients.

## Discussion

The use of SDD for infection control and AMR is a hotly contested topic revolving around the effect of SDD on AMR.^7 14^ Reduction in AMR has previously been reported in SDD trials in critically ill adults. By leveraging a pilot cluster randomised clinical trial,^15^ we were able to examine the microbiomes and resistomes of children treated in PICUs receiving SDD or standard infection control for the duration of mechanical ventilation.

We observed no difference in total AMR gene abundance between these groups. Of study-relevant AMR classes, only *ctx-M* beta-lactamases (one of the leading extended-spectrum beta-lactamases) were differentially increased at extubation in SC patients. This could suggest an important role for SDD in controlling the spread of this critically important class of antibiotic resistance genes.

Changes to the microbiome of critically ill children have previously been examined using 16S rRNA gene sequencing,^17 18 37^ but this is the first study to use metagenomic shotgun sequencing on longitudinal samples in the paediatric population in order to examine the AMR pool of the upper and lower GI microbiome.

Buelow *et al* 38 reported microbiome and resistome changes in a single adult patient receiving SDD-enhanced infection control using shotgun metagenomics. They reported a significant increase in tobramycin-modifying enzyme after 14 and 16 days of SDD^38^; this result was not observed in our study which had much shorter per-patient intervention duration. The highly individual effect of aminoglycoside resistance observed previously^38^ and the lack of significant change in AMR gene burden observed in our study would suggest that SDD is no worse than SC in driving AMR in PICUs; however, a sufficiently powered study is required to confirm this.

While many clinical trials and systematic reviews have reported the efficacy of SDD,^6 9 11 14 38–43^ the argument against the use of SDD in clinical practice persists.^38 44–46^ However, care must be taken when comparing the findings of our study with data from adult patient cohorts given the differences in microbiome composition and underlying morbidity between children and adults.

Certainly, our work highlights the importance of assessing microbiome recovery in any future definitive studies of SDD-enhanced infection control in critically ill children.

We found the microbiome richness of critically ill children to decrease with increased duration of stay in the PICU.^37^ A small decrease in alpha diversity was noted in the SDD-treated cohort at the time of extubation and in samples taken in convalescence. This is most likely due to the targeting of metabolic keystone species by both routinely administered antimicrobials as well as SDD.^47 48^ It would be remiss to assume that because SDD was designed to control the abundance of GNAR, that they are the only affected taxa.^49^

Previous studies of SDD in adults have reported reduced VAP and are variably associated with the reduction in HCAIs.^9 14 50^ Of the Gammaproteobacteria, the *Enterobacteriaceae* represented the largest proportion of gut-resident GNAR in our study. The proportion of *Enterobacteriaceae* in faecal samples was reduced after treatment with SDD compared with admission samples and post-treatment SC samples. From these data, we can conclude that SDD was effective in reducing susceptible GNARs in our patients. Previous work has reported the eradication of *E. coli* by days 9–10 of full SDD administration with intravenous third-generation cephalosporins.^51^ Our median time to extubation was 4–5 days at which point SDD was discontinued. In the study by Buelow *et al,* 51 an SDD administration time between 1 and 5 days coincided with relative proportions of *E. coli* 16S rRNA gene signal in critically ill adult patient samples that was comparable with healthy controls. This delayed reduction of bacterial burden may explain the presence of moderate levels of *Escherichia* in our extubation samples. The reduction in the median proportion of the *Enterobacteriaceae* at extubation was expected, but there was an unexpected increase in their median proportion in recovery samples, combined with an expansion in *Bifidobacterium* and a decrease in observed alpha diversity. The *Pseudomonadaceae* were not significantly affected by SDD treatment; however, a larger study is needed to confirm this effect.

It is well established that antimicrobials affect the microbiome.^52–55^ Chng *et al* 52 reported a sustained decrease in alpha diversity after the cessation of antimicrobial therapy and identified the absence of early and midpoint fermenter organisms^56 57^ generating metabolites used as an energy source by many gut health commensals.^52 58 59^ If slow-growing early and midpoint fermenters are adversely affected by SDD, they may be supplanted by less advantageous quick-replicating opportunistic pathogens, especially in developing microbiomes. While SDD may positively affect patients during their stay, from the data at hand, it could be inferred that SDD delays the recovery of the microbiome.

The AMR burden (measured in RPKM) in the faecal microbiome was similar between groups at admission and extubation. This suggests that SDD does not drive an increase in AMR spread. This is in concordance with reports from adult ICUs where SDD treatment coincided with reduced AMR in bacterial cultures during SDD.^9 42 60^ This change may be driven by an observed increase in the proportion of *Enterobacteriaceae.* RPKM was chosen over number of genes identified as it better reflects gene copy number in shotgun metagenomic data. The phylogenetic reclassification of *Enterobacteriaceae* by Adeolu *et al* 61 has removed genera with intrinsic colistin-resistant species such as *Hafnia*, *Morganella*, *Proteus*, *Providencia*, *Serratia* and *Yersinia* from the taxonomic family.

SDD has been shown to be effective in adult ICUs,^12 40 50^ and the PICnIC Study showed that SDD implementation across individual PICUs was feasible. As the microbiota of very young children is still developing and may not have a robust metabolic network in place, examining the long-term effect of SDD on the paediatric microbiome would be of great value.

### Limitations

As the first study to examine in detail the temporal impact of both critical illness and SDD on the microbiome, our study offers interesting insights and could inform the design of a larger surveillance study.

The PICnIC pilot trial was a scoping study and not powered to identify clinical significant effects of SDD on patients.^16^ This substudy was designed to gather baseline data to inform sample size for a larger multicentre clinical trial. A larger cohort with more frequent and longer-term post-treatment sampling would help determine the effects of SDD on the maturing microbiome.

Our study protocol omitted the administration of intravenous third-generation cephalosporins as the SC in UK PICUs is to administer a third-generation cephalosporin or suitable alternative drug upon admission to PICU if clinically required. Our patients all received a third-generation cephalosporin, or a broad-spectrum penicillin in combination with a beta-lactamase inhibitor. Our healthy controls were selected from a large cohort study conducted in the USA due to lack of available shotgun metagenomic data for UK and European children. The technical factors between our children and the US control children metagenomic sequencing may differ.

## Conclusion

SDD did not increase the AMR burden of patients at the end of their treatment but may extend the microbiota recovery period of patients. Our study observed that patient microbiomes were different during extubation and recovery. SDD patients had a greater abundance of Actinomycetota at extubation and reduced *Enterobacteriaceae*, while recovery SDD samples appeared high in Gram-negative organisms. More change was observed in the microbiome of SDD-treated patients than the SC group and is most likely due to the effects of SDD.

10.1136/gutjnl-2023-330851.supp3 Supplementary data

10.1136/gutjnl-2023-330851.supp4 Supplementary data

## Data Availability

Data are available in a public, open access repository. Data are available upon reasonable request. Sequencing data from the PICnIC-ARCTIC Study are available at https://www.ebi.ac.uk/ena/browser/view/PRJEB71687 (ERP156474). Sequencing data from baseline critically ill children are available at https://www.ebi.ac.uk/ena/browser/view/PRJEB71688 (ERP156475).
