# Supplementary material for: Short-duration selective decontamination of the digestive tract infection control does not contribute to increased antimicrobial resistance burden in a pilot cluster randomised trial (the ARCTIC Study)
Source: Gut. 2024 Jan 22;73(6):910–21. doi: 10.1136/gutjnl-2023-330851 (PMC11103307; doi:10.1136/gutjnl-2023-330851)
Supplement: Supplementary data [file gutjnl-2023-330851supp002.pdf]

SUPPLEMENTAL MATERIALS AND METHODS

Data Processing:

Sequences were quality trimmed using trim_galore v0.5.0 with default settings [1],and host sequences were removed using Bowtie2 v2.3.5.1 [2] and the GRCh38.p14 human reference genome [3]. Metagenomic composition was determined from cleaned reads using Kraken2 v2.0.9-beta [4] and the nt database (2023/05/02) [5].

Kraken2 was run with paired input sequences and confidence set to 0.1. Kraken data was compiled by identifying reads at each taxonomic level. Taxonomic classification was compiled at species level, with reads identified only at higher taxonomic levels being designated as "_unclassified". Only taxa with > 10 reads were included in further analysis.

AMR genes were identified from paired-end reads using ARIBA v2.14.6 and the CARD database v 3.2.7 [6,7]. ARIBA was run with an assembly threshold of 0.6 and a minimum id value of 80.

Statistical analysis was performed using R v4.2.0 [8]. Read counts mapping to host DNA, spurious taxa (plants, food, contaminants in negative controls) were removed from further analysis. After the removal of confounding counts, bacterial sequence counts were normalised to reads per million reads. Using the assumption in which sequencing reads are modelled along a Gamma distribution, unclassified reads of higher taxonomic level were proportionally distributed across their subdivisions.

To perform analysis of the alpha- and beta-diversity of samples, taxonomy was aggregated at species level. Using vegan v 2.6-2 [9], and the diversity function, we calculated Shannon's Index for each sample. Shannon's index was visualised by

creating Tukey's Box and Whisker plot using the Tidyverse package ggplot2 [10] with the ploting function geom_boxplot. To identify changed between groups, paired Wilcoxon tests were used for intra-treatment samples, and unpaired test for inter-treatment comparisons. Comparisons were made between the following groups SDD-1:SDD-2, SDD-1:SDD-3, SDD-1:SC-1, SDD-2:SDD-3, SDD-2:SC-2, SDD-3:SC-3, SC-1:SC-2,SC-1:SC-3, and SC-2:SC-3. The resultant p-values for the repeated Wilcoxon tests were corrected using Benjamini and Hochberg's False Discovry Rate (FDR) correction as applied by p.adjust function of base R.

The Chao1 index was calculated by using the estimateR function of vegan using the floor function on the calculated RPMR value. Visualization and multiple comparisons were performed as for Shannon's Index.

Beta-distances were calculated against the table of species normalised as RPRM using the vegdist function of vegan with Bray-Curtis distances. To visualise the clustering, we perfomed nMDS using the metaMDS function of vegan using 200 iterations, and vectors 1 and 2 were plotted using ggplot and the geom_point function. The analysis of clustering was performed using Adonis2. To identify separation between groups, Adonis2 was performed for each pair-wise comparison of the above list, and between all timepoints of treatment groups. When comparisons were from a single treatment group (paired or the entire group), Adonis2 was stratified by patient identifier. The resulting p-values from pair-wise and treatment-groupwise Adonis2 calculations were normalised using FDR.

To visualize the composition of bacterial taxonomy, taxonomic data was aggregated at a Genus level. From the RPMR normalised table, counts were aggregated using the aggregate function of the stats package of R. To identify the 10 most abundant taxa in SDD patients, the median counts of each bacterial genera were calculated

using the summarise function of dplyr (Tidyverse suite). Median was chosen as our read-count data is not normally distributed. To identify the 10 most abundant genera in the SC group, the median count for each taxa was calculated, the list of 10 taxa from SDD patients was removed from list of medians, and the next ten most abundant bacteria were selected. All other taxa were aggregated into "Other" to simplify graphing. Standard column graphs were created using ggplot2 and the geom_col function, setting the position to "fill". For continuity and ease of interpretation, the taxa selected from admission samples was used to observe the changes across timepoints.

For the alluvial plots, we used the ggplot2 extension package ggalluvial, and taxonomy from the above boxplots. Alluvial plots were included to highlight changes in the microbiome composition throughout each treatment.

The software package MaAsLin2 v 1.10.0 [11] was used to perform further interrogations of the microbiomes. To analyse time dependent change in groups, patient ID was set as a factor and applied as a random effect, sampling number, weight and age were treated as fixed effects and a minimum prevalence of 0.4 was applied. To compare treatment dependent changes, treatment group, weight and age were treated as fixed effects and a minimum prevalence of 0.4 was applied. Multiple comparisons were corrected using FDR, and all other settings were used as default. Where the difference between more than four taxa was identified by MaAsLin2, the coefficient of change was plotted as horizontal column graph. For interpretation, the absolute value of the coefficient was plotted with the signed direction of change illustrated by column colour. We chose to set both α and the adjusted-α at a rate of 1 in 20.

To compare the top 20 taxa between groups at each timepoint, we calculated the difference for each taxa using a Kruskal-Wallis test, as the KW test is a generalised Mann-Whitney U test. The p-value for each test was adjusted after calculation by applying the FDR correction, with q-values reported.

Analysis of resistance genes:

To focus the analysis of AMR, genes involved in resistance to non-clinically relevant compounds such as heavy metals, detergents, cleaning compounds and tetracyclines were excluded. Statistical analysis and visualization of AMR genes followed the methods outlined previously.

Genes detected with ARIBA were compiled for each patient and measured in Reads per Kilobase$_{gene}$ per Megabase of sequencing (RPKM).

Sequencing and analysis statistics of PICNIC patients is available in Supplementary Table 1

Sequencing and analysis statistics from samples obtained from described by Clark et al [12] are available in Supplementary Table 2.

1 Krueger F. Trim Galore. 2022. https://github.com/FelixKrueger/TrimGalore (accessed 2 November 2022)

2 Langmead B, Salzberg SL. Fast gapped-read alignment with Bowtie 2. *Nat Methods*. 2012;9:357–9.

3 GRCh38.p14 - hg38 - Genome - Assembly - NCBI. https://www.ncbi.nlm.nih.gov/assembly/GCF_000001405.40/ (accessed 2 November 2022)

4 Wood DE, Lu J, Langmead B. Improved metagenomic analysis with Kraken 2. *Genome Biology*. 2019;20:257.

5 Index zone by BenLangmead. https://benlangmead.github.io/aws-indexes/k2 (accessed 16 November 2023)

6 Bonin N, Doster E, Worley H, *et al.* MEGARes and AMR++, v3.0: an updated comprehensive database of antimicrobial resistance determinants and an improved

software pipeline for classification using high-throughput sequencing. *Nucleic Acids Res*. 2023;51:D744–52.

7 Jia B, Raphenya AR, Alcock B, *et al.* CARD 2017: expansion and model-centric curation of the comprehensive antibiotic resistance database. *Nucleic Acids Res*. 2017;45:D566–73.

8 R: A language and environment for statistical computing. 2022. https://www.R-project.org/

9 Oksanen J, Simpson GL, Blanchet FG, *et al.* vegan: Community Ecology Package. 2022. https://CRAN.R-project.org/package=vegan (accessed 2 November 2022)

10 Wickham H, Averick M, Bryan J, *et al.* Welcome to the Tidyverse. *Journal of Open Source Software*. 2019;4:1686.

11 Mallick H, Rahnavard A, McIver LJ, *et al.* Multivariable association discovery in population-scale meta-omics studies. *PLOS Computational Biology*. 2021;17:e1009442.

12 Clark JA, Conway Morris A, Curran MD, *et al.* The rapid detection of respiratory pathogens in critically ill children. *Crit Care*. 2023;27:11.
