# Supplementary material for: Short-duration selective decontamination of the digestive tract infection control does not contribute to increased antimicrobial resistance burden in a pilot cluster randomised trial (the ARCTIC Study)
Source: Gut. 2024 Jan 22;73(6):910–21. doi: 10.1136/gutjnl-2023-330851 (PMC11103307; doi:10.1136/gutjnl-2023-330851)
Supplement: Supplementary data [file gutjnl-2023-330851supp001.pdf]

Figure S1

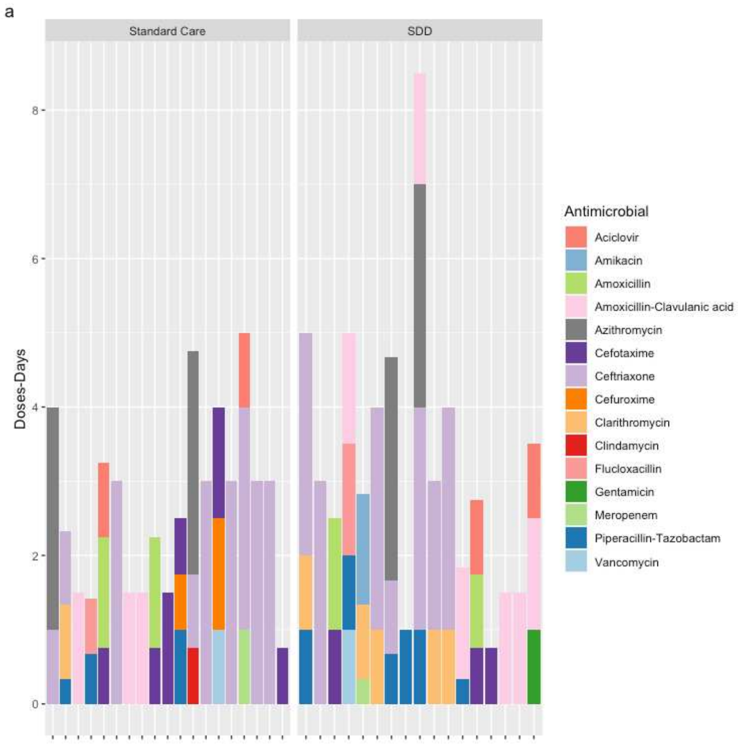
a
Standard Care
SDD
Doses-Days
0
2
4
6
8
Antimicrobial
Aciclovir
Amikacin
Amoxicillin
Amoxicillin-Clavulanic acid
Azithromycin
Cefotaxime
Ceftriaxone
Cefuroxime
Clarithromycin
Clindamycin
Flucloxacillin
Gentamicin
Meropenem
Piperacillin-Tazobactam
Vancomycin

BMJ Publishing Group Limited (BMJ) disclaims all liability and responsibility arising from any reliance placed on this supplemental material which has been supplied by the author(s)

Figure S1: Antimicrobial use in patient cohort, and comparison of healthy UK children to healthy US children. A)Antimicrobial use for children admitted to PICU. Antimicrobial use was scaled to doses (administered)/doses(day) (age adjusted based on BNFC guidelines).

Figure S2

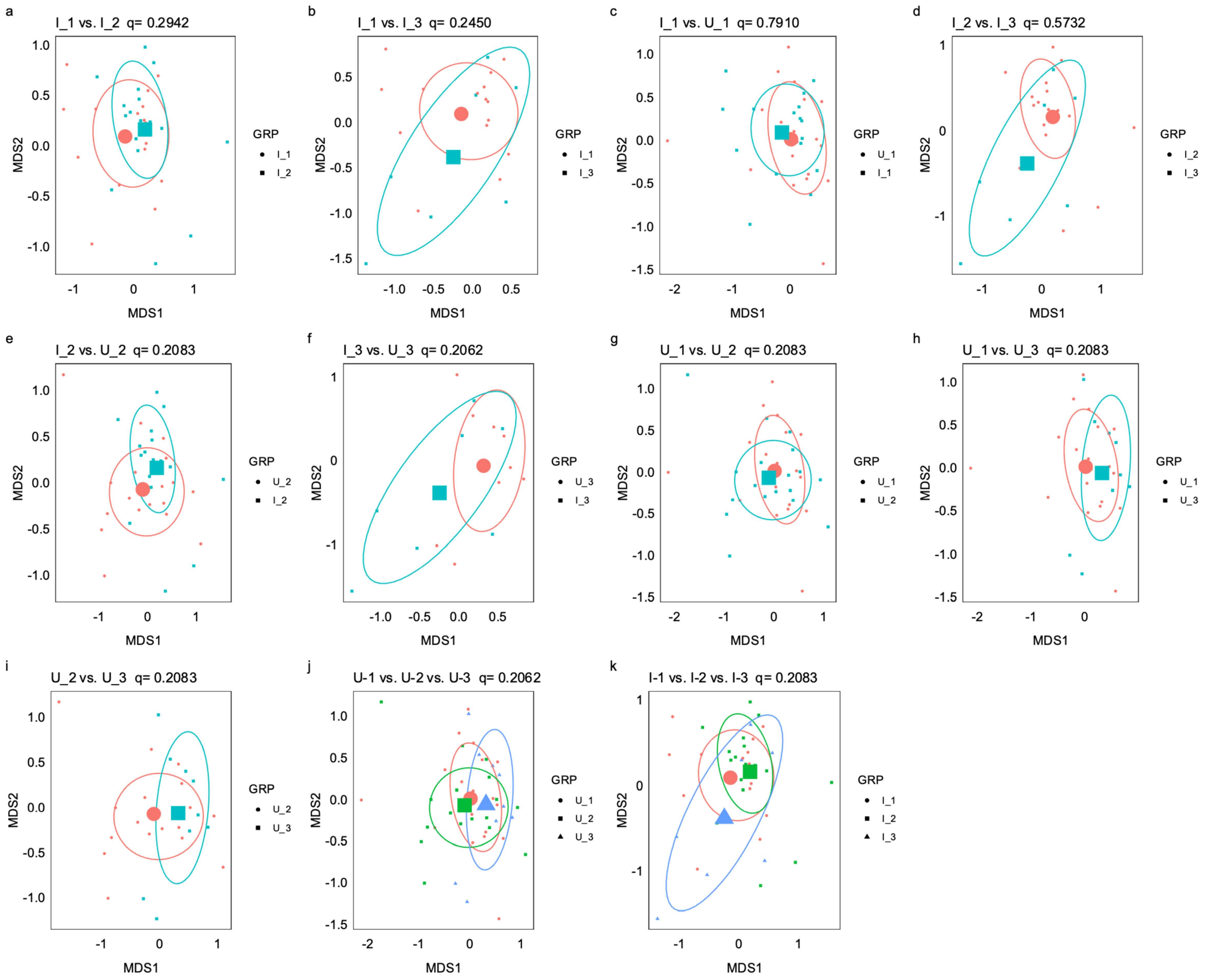
a
I_1 vs. I_2 q= 0.2942
b
I_1 vs. I_3 q= 0.2450
c
I_1 vs. U_1 q= 0.7910
d
I_2 vs. I_3 q= 0.5732
e
I_2 vs. U_2 q= 0.2083
f
I_3 vs. U_3 q= 0.2062
g
U_1 vs. U_2 q= 0.2083
h
U_1 vs. U_3 q= 0.2083
i
U_2 vs. U_3 q= 0.2083
j
U-1 vs. U-2 vs. U-3 q= 0.2062
k
I-1 vs. I-2 vs. I-3 q= 0.2083
MDS1
MDS2
GRP

Figure S2: Pair- and treatment group-wise comparisons of nMDS clustering. Q values represent p values adjusted for multiple comparisons using the FDR correction.

Figure S3

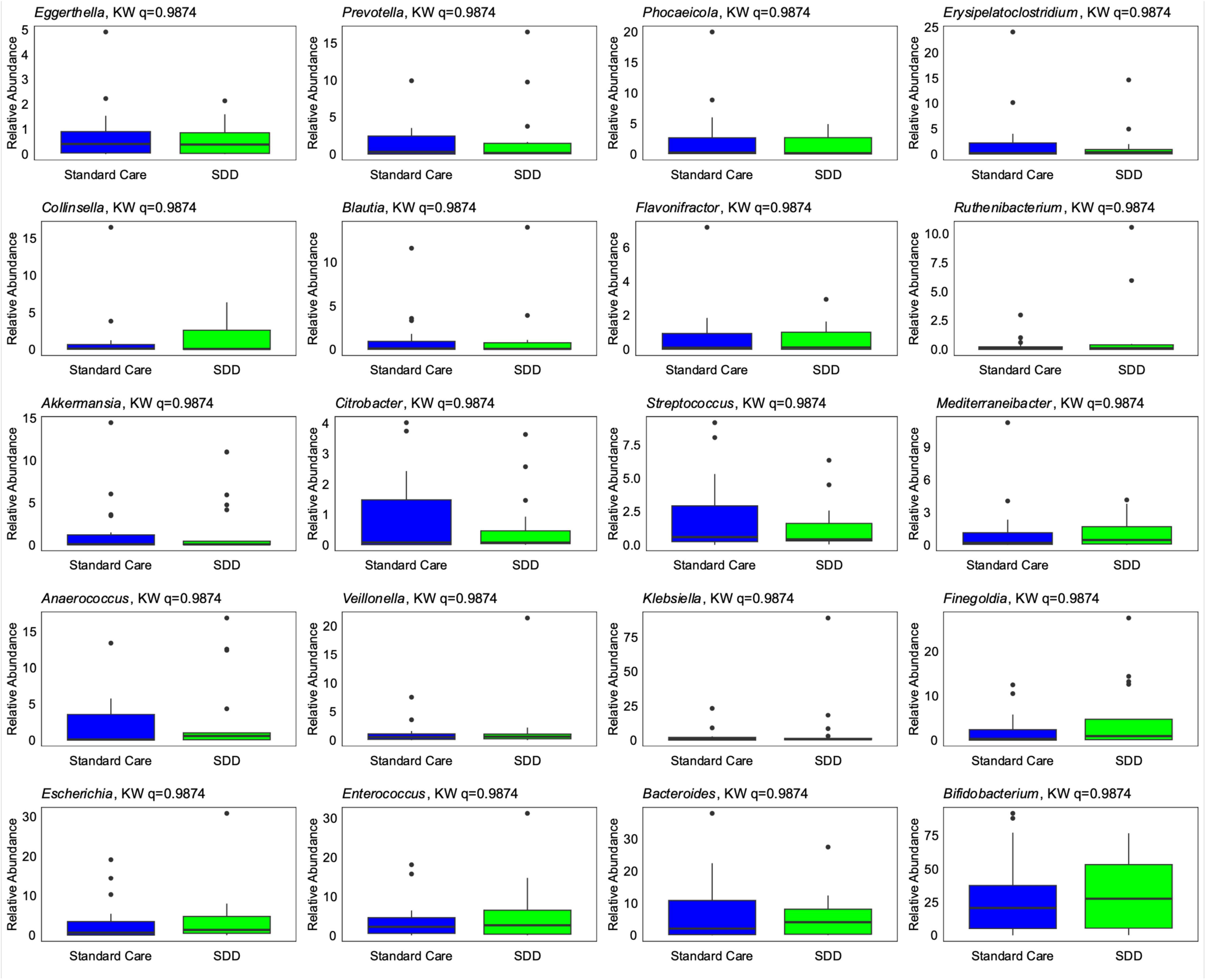
Eggerthella, KW q=0.9874
Prevotella, KW q=0.9874
Phocaeicola, KW q=0.9874
Erysipelatoclostridium, KW q=0.9874
Collinsella, KW q=0.9874
Blautia, KW q=0.9874
Flavonifractor, KW q=0.9874
Ruthenibacterium, KW q=0.9874
Akkermansia, KW q=0.9874
Citrobacter, KW q=0.9874
Streptococcus, KW q=0.9874
Mediterraneibacter, KW q=0.9874
Anaerococcus, KW q=0.9874
Veillonella, KW q=0.9874
Klebsiella, KW q=0.9874
Finegoldia, KW q=0.9874
Escherichia, KW q=0.9874
Enterococcus, KW q=0.9874
Bacteroides, KW q=0.9874
Bifidobacterium, KW q=0.9874
Relative Abundance
Standard Care
SDD

Figure S3: Admission lower gastrointestinal tract microbiota . A comparison of the 20 microbiota identified in Figure 2. Total statistical difference calculated by Kruskal-Wallis test. Multiple tests corrected by FDR.

Figure S4

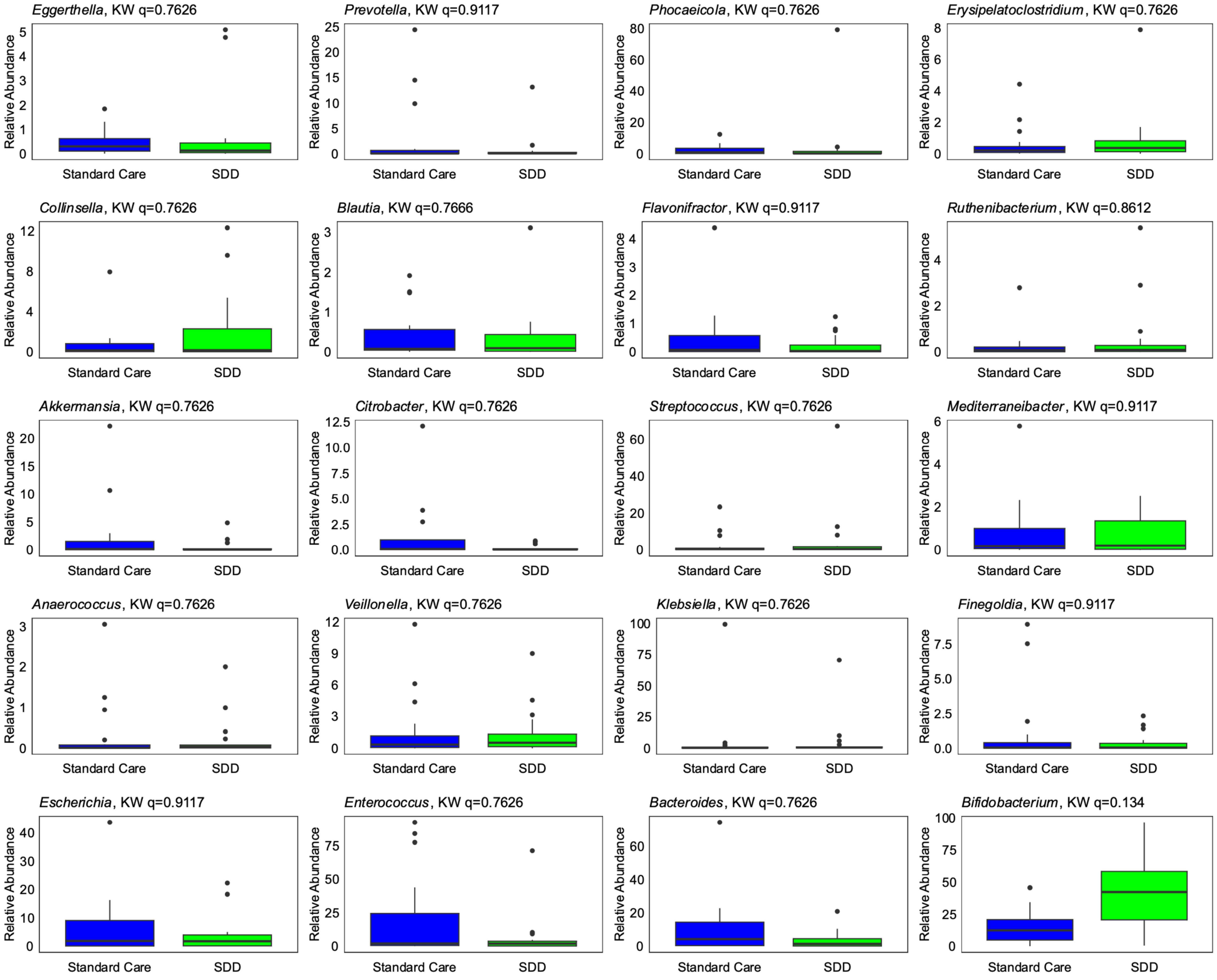
Eggerthella, KW q=0.7626
Prevotella, KW q=0.9117
Phocaeicola, KW q=0.7626
Erysipelatoclostridium, KW q=0.7626
Collinsella, KW q=0.7626
Blautia, KW q=0.7666
Flavonifractor, KW q=0.9117
Ruthenibacterium, KW q=0.8612
Akkermansia, KW q=0.7626
Citrobacter, KW q=0.7626
Streptococcus, KW q=0.7626
Mediterraneibacter, KW q=0.9117
Anaerococcus, KW q=0.7626
Veillonella, KW q=0.7626
Klebsiella, KW q=0.7626
Finegoldia, KW q=0.9117
Escherichia, KW q=0.9117
Enterococcus, KW q=0.7626
Bacteroides, KW q=0.7626
Bifidobacterium, KW q=0.134
Relative Abundance
Standard Care
SDD

Figure S4 : Extubation lower gastrointestinal tract microbiota. A comparison of the 20 microbiota identified in Figure 2. Total statistical difference calculated by Kruskal-Wallis test. Multiple tests corrected by FDR.

Figure S5

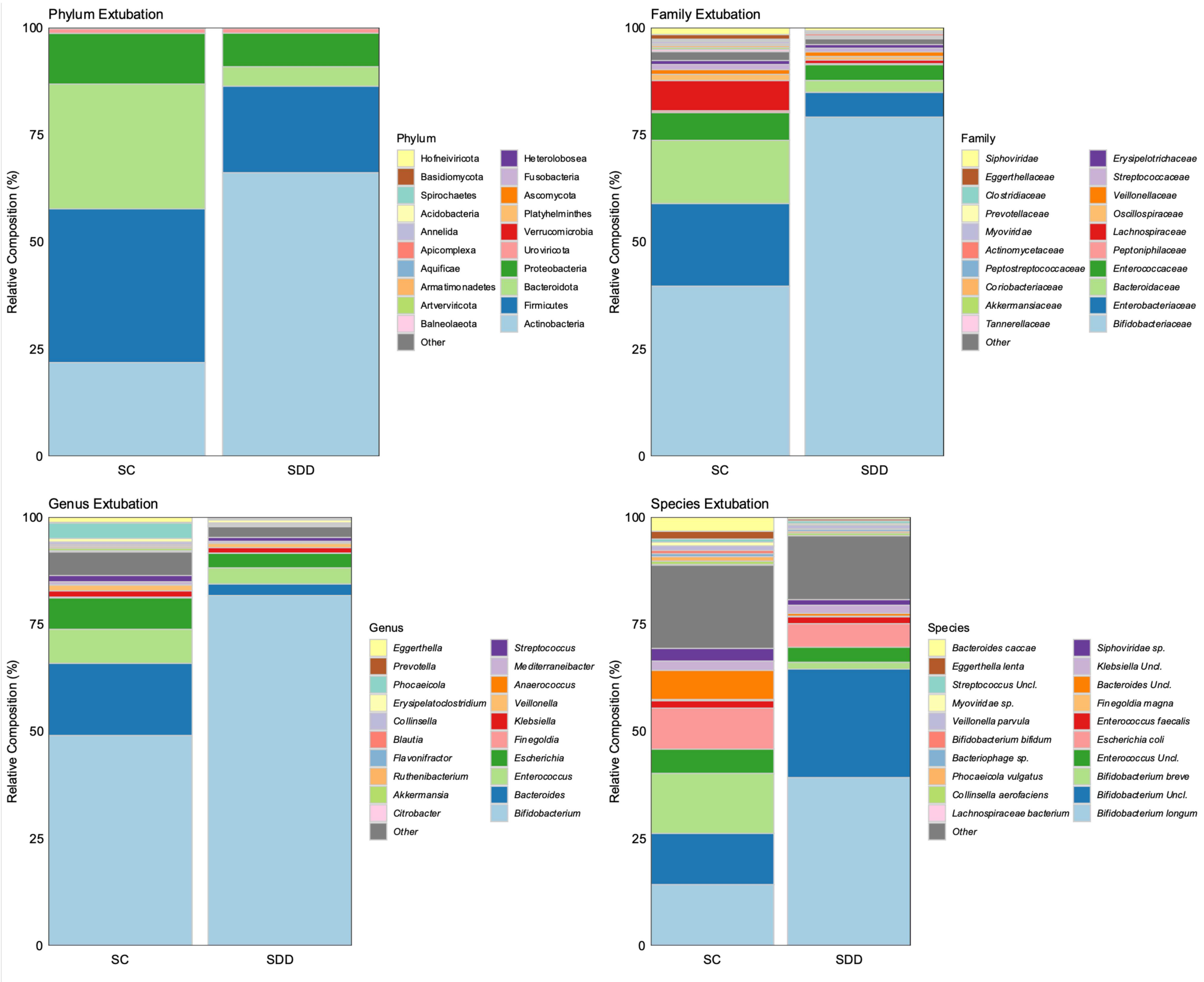
Phylum Extubation
Relative Composition (%)
0
25
50
75
100
SC
SDD
Phylum
Hofneiviricota
Basidiomycota
Spirochaetes
Acidobacteria
Annelida
Apicomplexa
Aquificae
Armatimonadetes
Artverviricota
Balneolaeota
Other
Heterolobosea
Fusobacteria
Ascomycota
Platyhelminthes
Verrucomicrobia
Uroviricota
Proteobacteria
Bacteroidota
Firmicutes
Actinobacteria
Family Extubation
Family
Siphoviridae
Eggerthellaceae
Clostridiaceae
Prevotellaceae
Myoviridae
Actinomycetaceae
Peptostreptococcaceae
Coriobacteriaceae
Akkermansiaceae
Tannerellaceae
Other
Erysipelotrichaceae
Streptococcaceae
Veillonellaceae
Oscillospiraceae
Lachnospiraceae
Peptoniphilaceae
Enterococcaceae
Bacteroidaceae
Enterobacteriaceae
Bifidobacteriaceae
Genus Extubation
Genus
Eggerthella
Prevotella
Phocaeicola
Erysipelatoclostridium
Collinsella
Blautia
Flavonifractor
Ruthenibacterium
Akkermansia
Citrobacter
Other
Streptococcus
Mediterraneibacter
Anaerococcus
Veillonella
Klebsiella
Finegoldia
Escherichia
Enterococcus
Bacteroides
Bifidobacterium
Species Extubation
Species
Bacteroides caccae
Eggerthella lenta
Streptococcus Uncl.
Myoviridae sp.
Veillonella parvula
Bifidobacterium bifidum
Bacteriophage sp.
Phocaeicola vulgatus
Collinsella aerofaciens
Lachnospiraceae bacterium
Other
Siphoviridae sp.
Klebsiella Uncl.
Bacteroides Uncl.
Finegoldia magna
Enterococcus faecalis
Escherichia coli
Enterococcus Uncl.
Bifidobacterium breve
Bifidobacterium Uncl.
Bifidobacterium longum

BMJ Publishing Group Limited (BMJ) disclaims all liability and responsibility arising from any reliance placed on this supplemental material which has been supplied by the author(s)

Figure S5: Comparison of extubation microbiomes at multiple taxonomic levels.

Figure S6

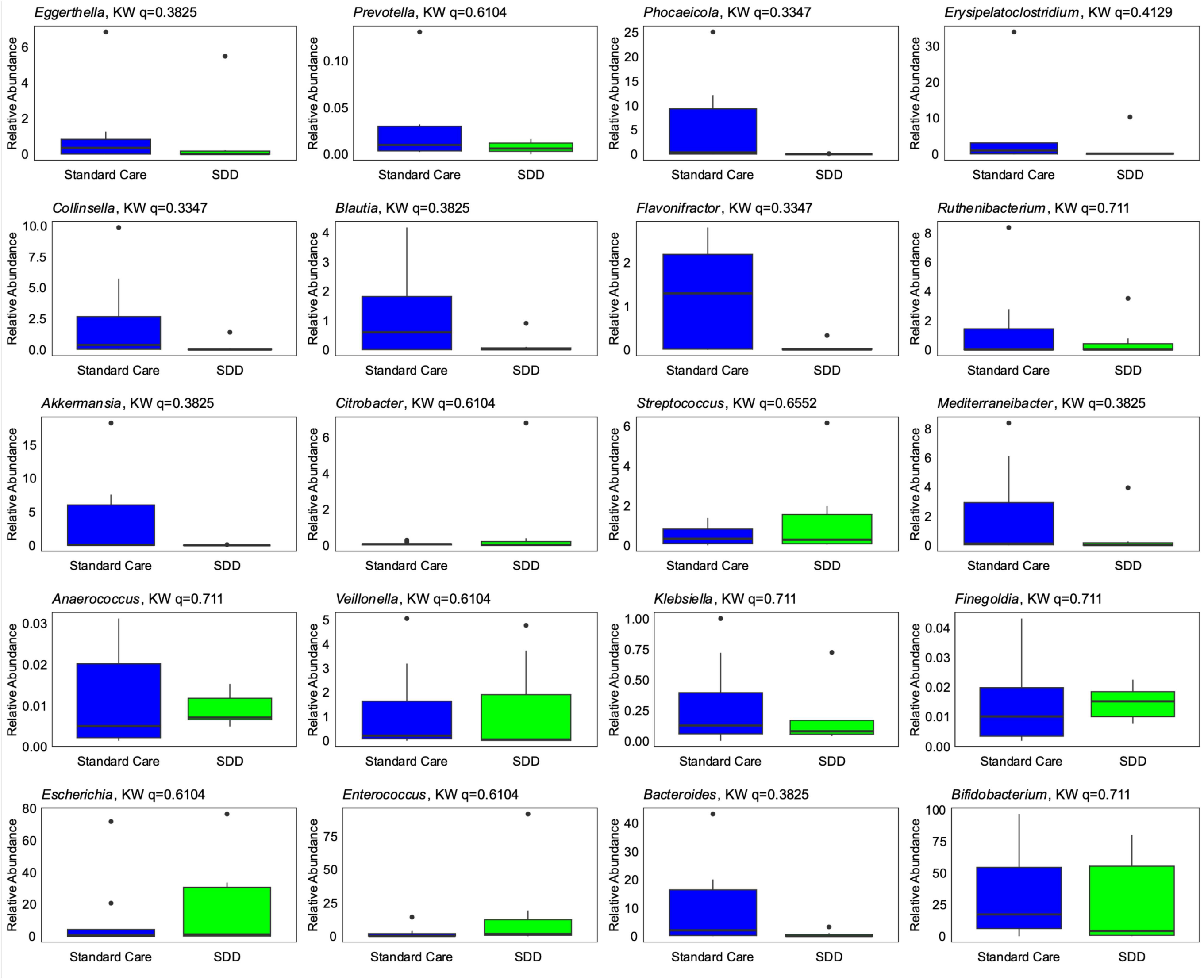

Eggerthella, KW q=0.3825
Prevotella, KW q=0.6104
Phocaeicola, KW q=0.3347
Erysipelatoclostridium, KW q=0.4129
Collinsella, KW q=0.3347
Blautia, KW q=0.3825
Flavonifractor, KW q=0.3347
Ruthenibacterium, KW q=0.711
Akkermansia, KW q=0.3825
Citrobacter, KW q=0.6104
Streptococcus, KW q=0.6552
Mediterraneibacter, KW q=0.3825
Anaerococcus, KW q=0.711
Veillonella, KW q=0.6104
Klebsiella, KW q=0.711
Finegoldia, KW q=0.711
Escherichia, KW q=0.6104
Enterococcus, KW q=0.6104
Bacteroides, KW q=0.3825
Bifidobacterium, KW q=0.711
Relative Abundance
Standard Care
SDD

Figure S6 : Recovery lower gastrointestinal tract microbiota. A comparison of the 20 microbiota identified in Figure 2. Total statistical difference calculated by Kruskal-Wallis test. Multiple tests corrected by FDR.

Figure S7

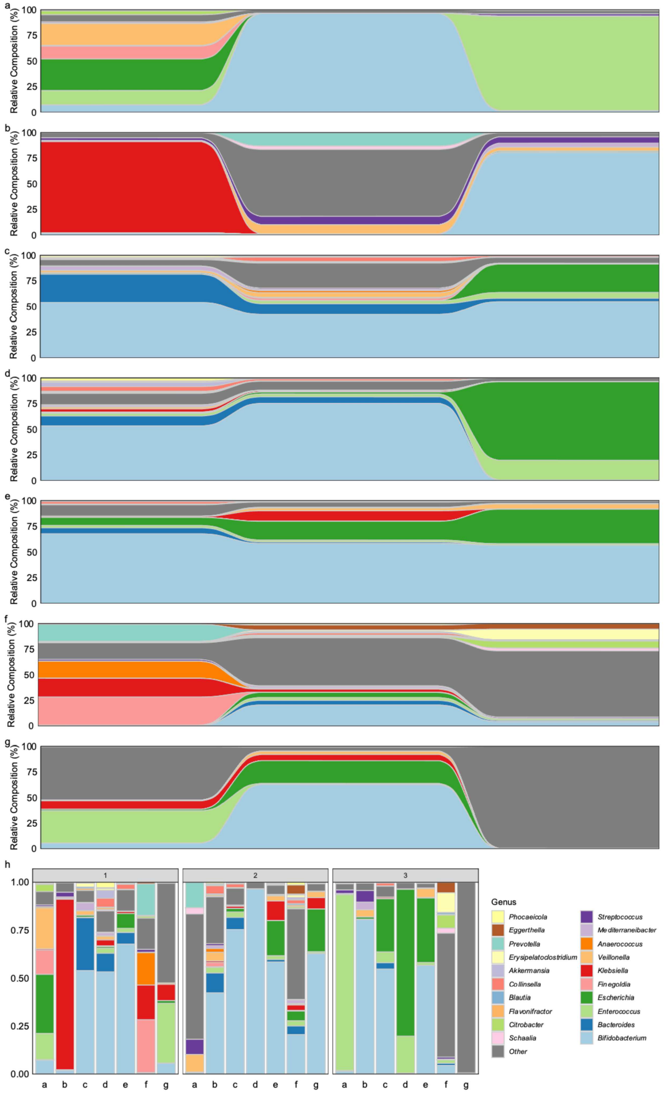
a
b
c
d
e
f
g
h
Relative Composition (%)
0
25
50
75
100
1
2
3
1.00
0.75
0.50
0.25
0.00
a b c d e f g
Genus
Phocaeicola
Eggerthella
Prevotella
Erysipelatoclostridium
Akkermansia
Collinsella
Blautia
Flavonifractor
Citrobacter
Schaalia
Other
Streptococcus
Mediterraneibacter
Anaerococcus
Veillonella
Klebsiella
Finegoldia
Escherichia
Enterococcus
Bacteroides
Bifidobacterium

Figure S7: Compositional change of SDD patients. A-G) Alluvial plots identifying compositional change at Genus level in the seven SDD patients returning a recovery sample.

Figure S8

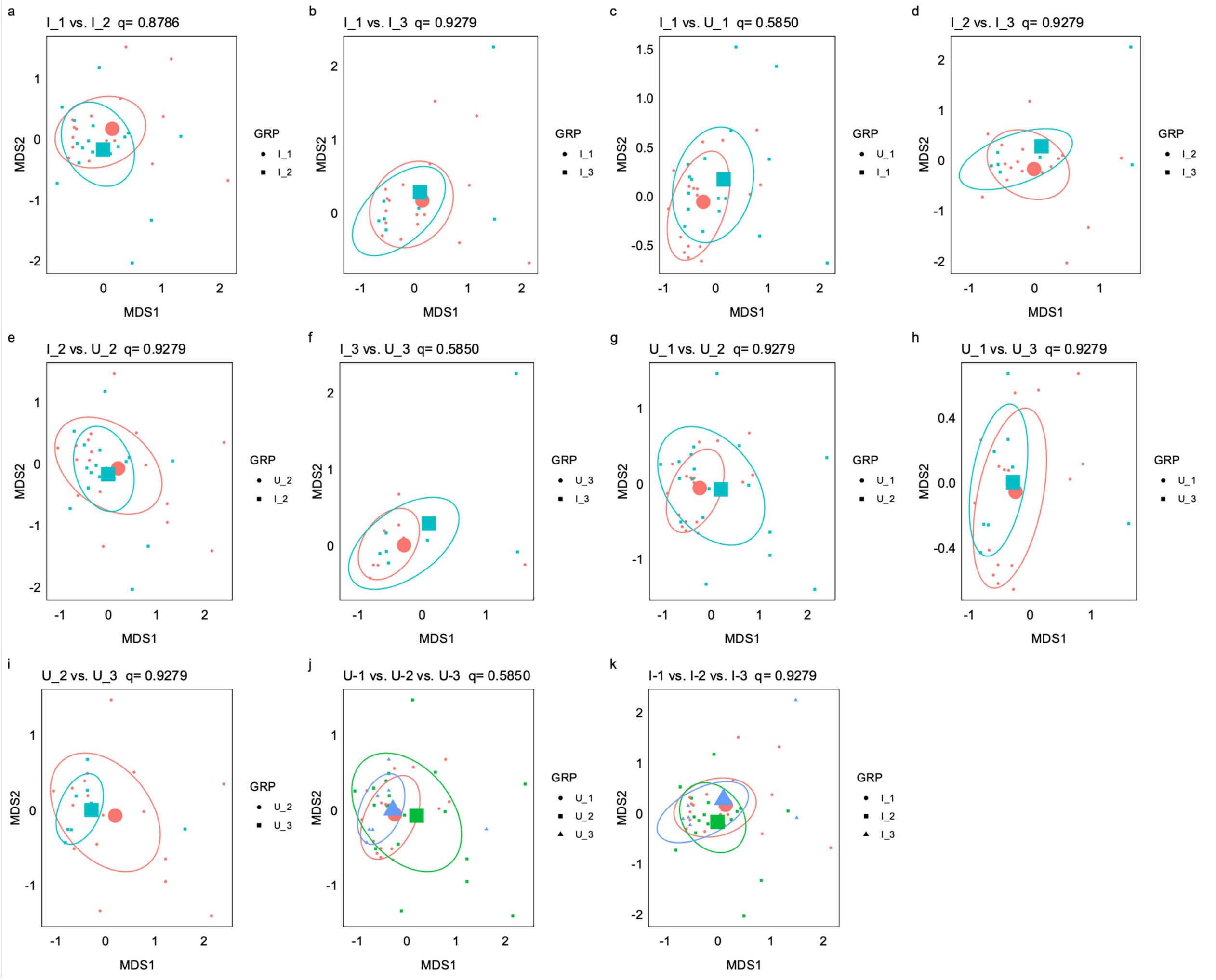
a
I_1 vs. I_2 q= 0.8786
b
I_1 vs. I_3 q= 0.9279
c
I_1 vs. U_1 q= 0.5850
d
I_2 vs. I_3 q= 0.9279
e
I_2 vs. U_2 q= 0.9279
f
I_3 vs. U_3 q= 0.5850
g
U_1 vs. U_2 q= 0.9279
h
U_1 vs. U_3 q= 0.9279
i
U_2 vs. U_3 q= 0.9279
j
U-1 vs. U-2 vs. U-3 q= 0.5850
k
I-1 vs. I-2 vs. I-3 q= 0.9279
MDS1
MDS2
GRP

Figure S8: pair and treatment group-wise comparisons of nMDS clustering. Q values represent p values adjusted for multiple comparisons using the FDR correction.

Figure S9

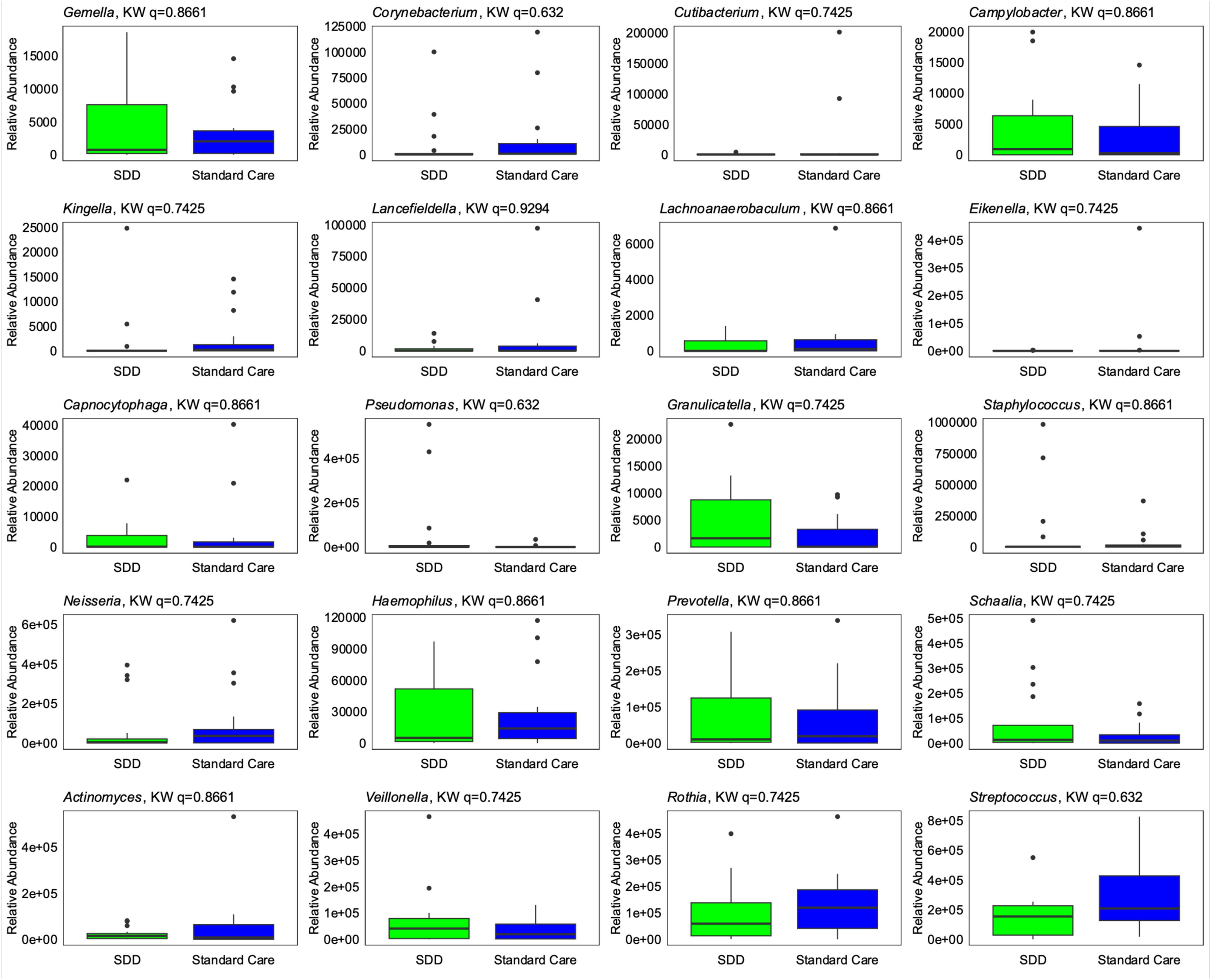
Gemella, KW q=0.8661
Corynebacterium, KW q=0.632
Cutibacterium, KW q=0.7425
Campylobacter, KW q=0.8661
Kingella, KW q=0.7425
Lancefieldella, KW q=0.9294
Lachnoanaerobaculum, KW q=0.8661
Eikenella, KW q=0.7425
Capnocytophaga, KW q=0.8661
Pseudomonas, KW q=0.632
Granulicatella, KW q=0.7425
Staphylococcus, KW q=0.8661
Neisseria, KW q=0.7425
Haemophilus, KW q=0.8661
Prevotella, KW q=0.8661
Schaalia, KW q=0.7425
Actinomyces, KW q=0.8661
Veillonella, KW q=0.7425
Rothia, KW q=0.7425
Streptococcus, KW q=0.632
Relative Abundance
SDD
Standard Care

BMJ Publishing Group Limited (BMJ) disclaims all liability and responsibility arising from any reliance placed on this supplemental material which has been supplied by the author(s)

Figure S9: Admission oropharyngeal microbiota . A comparison of the 20 microbiota identified in Figure 4. Total statistical difference calculated by Kruskal-Wallis test. Multiple tests corrected by FDR.

Figure S10

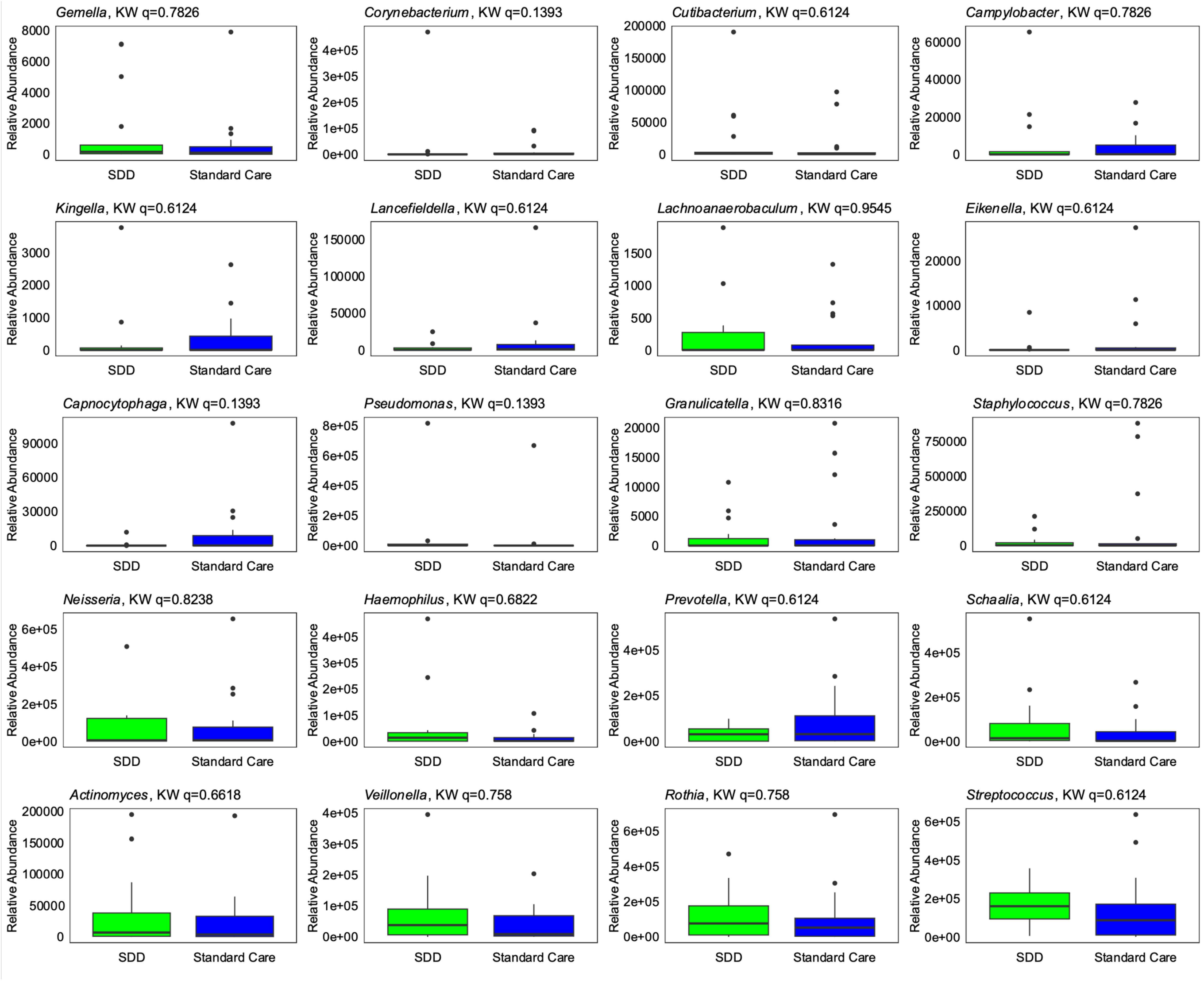
Gemella, KW q=0.7826
Corynebacterium, KW q=0.1393
Cutibacterium, KW q=0.6124
Campylobacter, KW q=0.7826
Kingella, KW q=0.6124
Lancefieldella, KW q=0.6124
Lachnoanaerobaculum, KW q=0.9545
Eikenella, KW q=0.6124
Capnocytophaga, KW q=0.1393
Pseudomonas, KW q=0.1393
Granulicatella, KW q=0.8316
Staphylococcus, KW q=0.7826
Neisseria, KW q=0.8238
Haemophilus, KW q=0.6822
Prevotella, KW q=0.6124
Schaalia, KW q=0.6124
Actinomyces, KW q=0.6618
Veillonella, KW q=0.758
Rothia, KW q=0.758
Streptococcus, KW q=0.6124
Relative Abundance
SDD
Standard Care

BMJ Publishing Group Limited (BMJ) disclaims all liability and responsibility arising from any reliance placed on this supplemental material which has been supplied by the author(s)

Figure S10: Extubation oropharyngeal microbiota . A comparison of the 20 microbiota identified in Figure 4. Total statistical difference calculated by Kruskal-Wallis test. Multiple tests corrected by FDR.

Figure S11

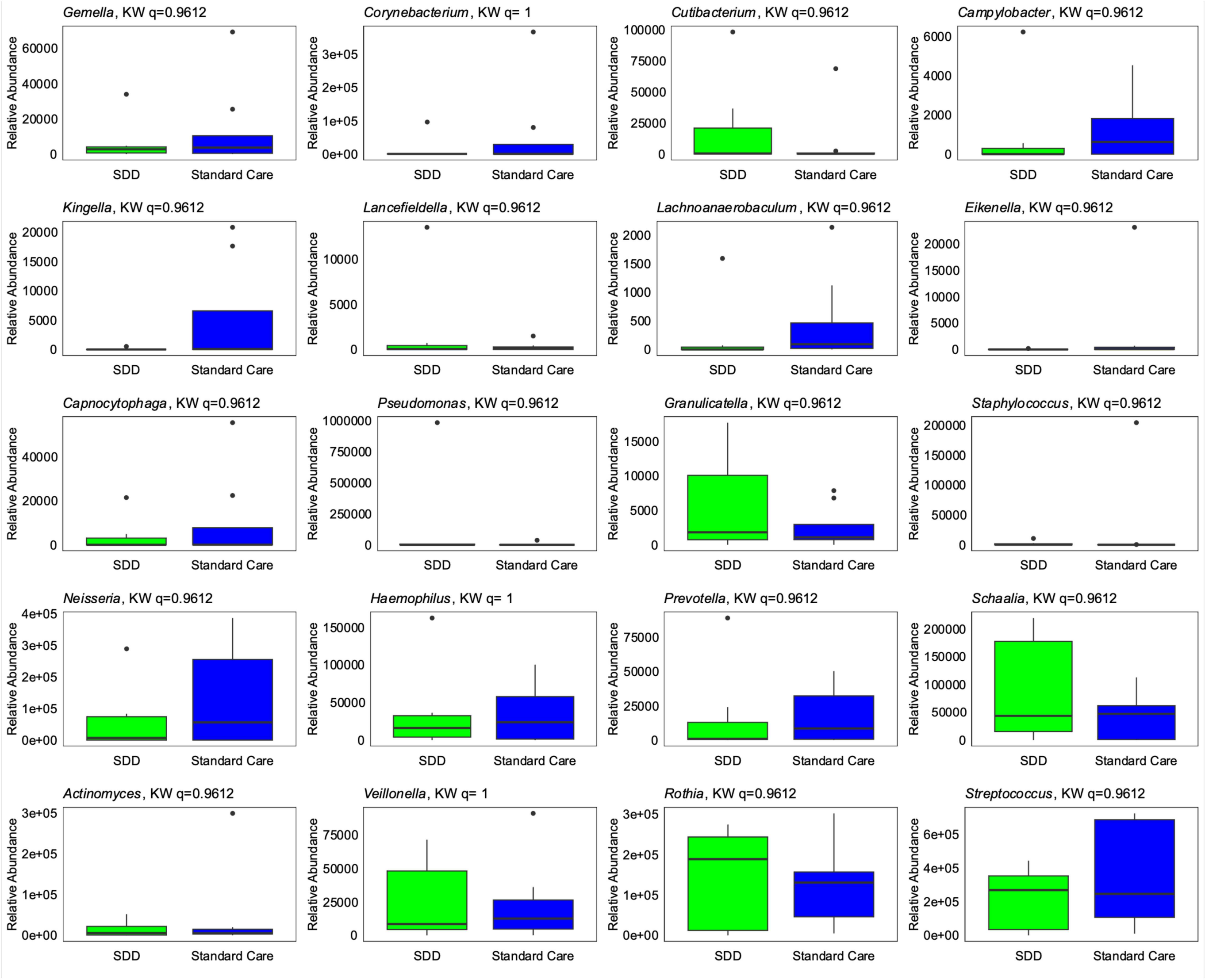

Gemella, KW q=0.9612
Corynebacterium, KW q= 1
Cutibacterium, KW q=0.9612
Campylobacter, KW q=0.9612
Kingella, KW q=0.9612
Lancefieldella, KW q=0.9612
Lachnoanaerobaculum, KW q=0.9612
Eikenella, KW q=0.9612
Capnocytophaga, KW q=0.9612
Pseudomonas, KW q=0.9612
Granulicatella, KW q=0.9612
Staphylococcus, KW q=0.9612
Neisseria, KW q=0.9612
Haemophilus, KW q= 1
Prevotella, KW q=0.9612
Schaalia, KW q=0.9612
Actinomyces, KW q=0.9612
Veillonella, KW q= 1
Rothia, KW q=0.9612
Streptococcus, KW q=0.9612
Relative Abundance
SDD
Standard Care

Figure S11: Recovery oropharyngeal microbiota . A comparison of the 20 microbiota identified in Figure 4. Total statistical difference calculated by Kruskal-Wallis test. Multiple tests corrected by FDR.

Figure S12

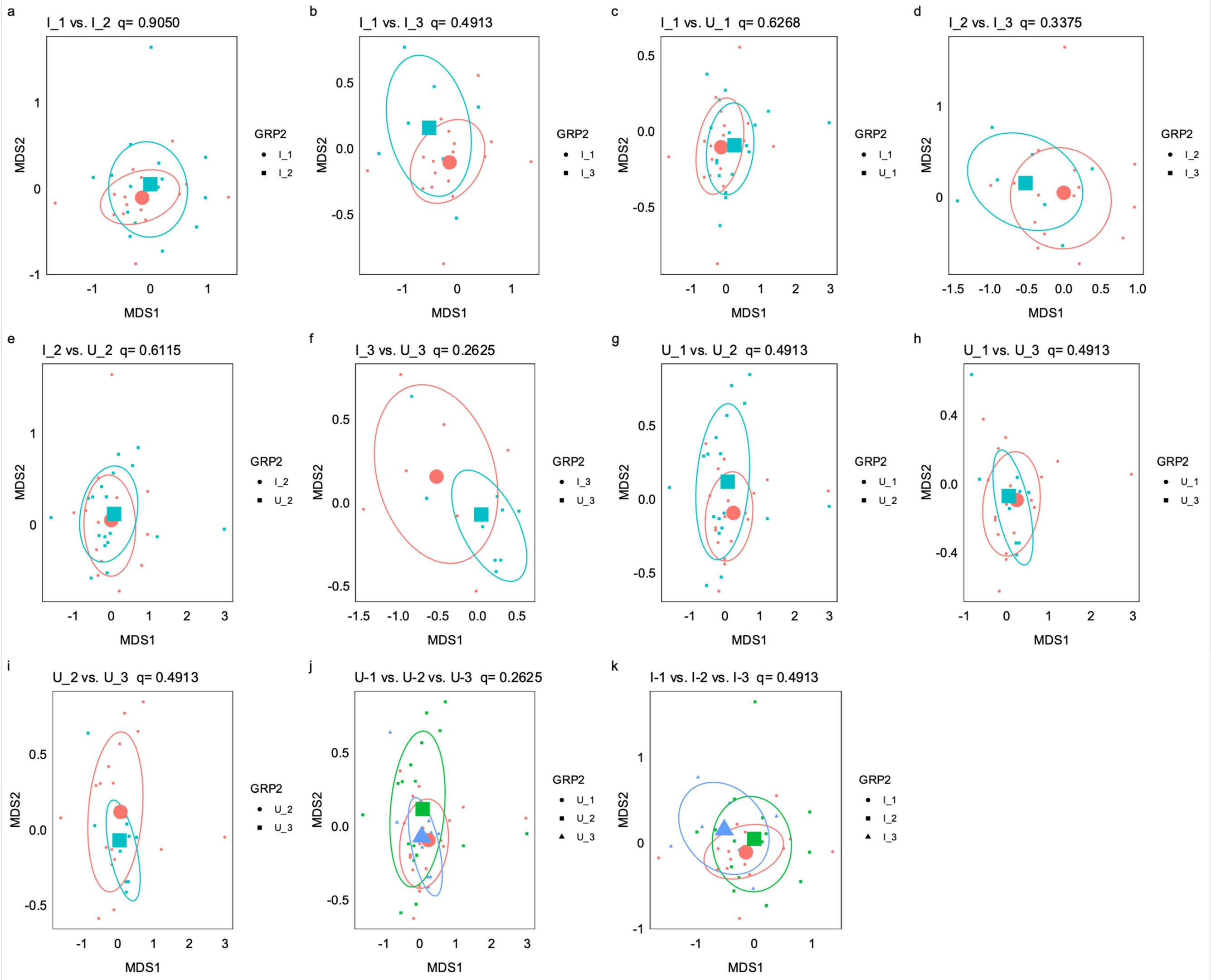
a
I_1 vs. I_2 q= 0.9050
b
I_1 vs. I_3 q= 0.4913
c
I_1 vs. U_1 q= 0.6268
d
I_2 vs. I_3 q= 0.3375
e
I_2 vs. U_2 q= 0.6115
f
I_3 vs. U_3 q= 0.2625
g
U_1 vs. U_2 q= 0.4913
h
U_1 vs. U_3 q= 0.4913
i
U_2 vs. U_3 q= 0.4913
j
U-1 vs. U-2 vs. U-3 q= 0.2625
k
I-1 vs. I-2 vs. I-3 q= 0.4913
MDS1
MDS2
GRP2

Figure S12: Pair and treatment group-wise comparisons of nMDS clustering for Faecal AMR. Q values represent p values adjusted for multiple comparisons using the FDR correction.

Figure S13

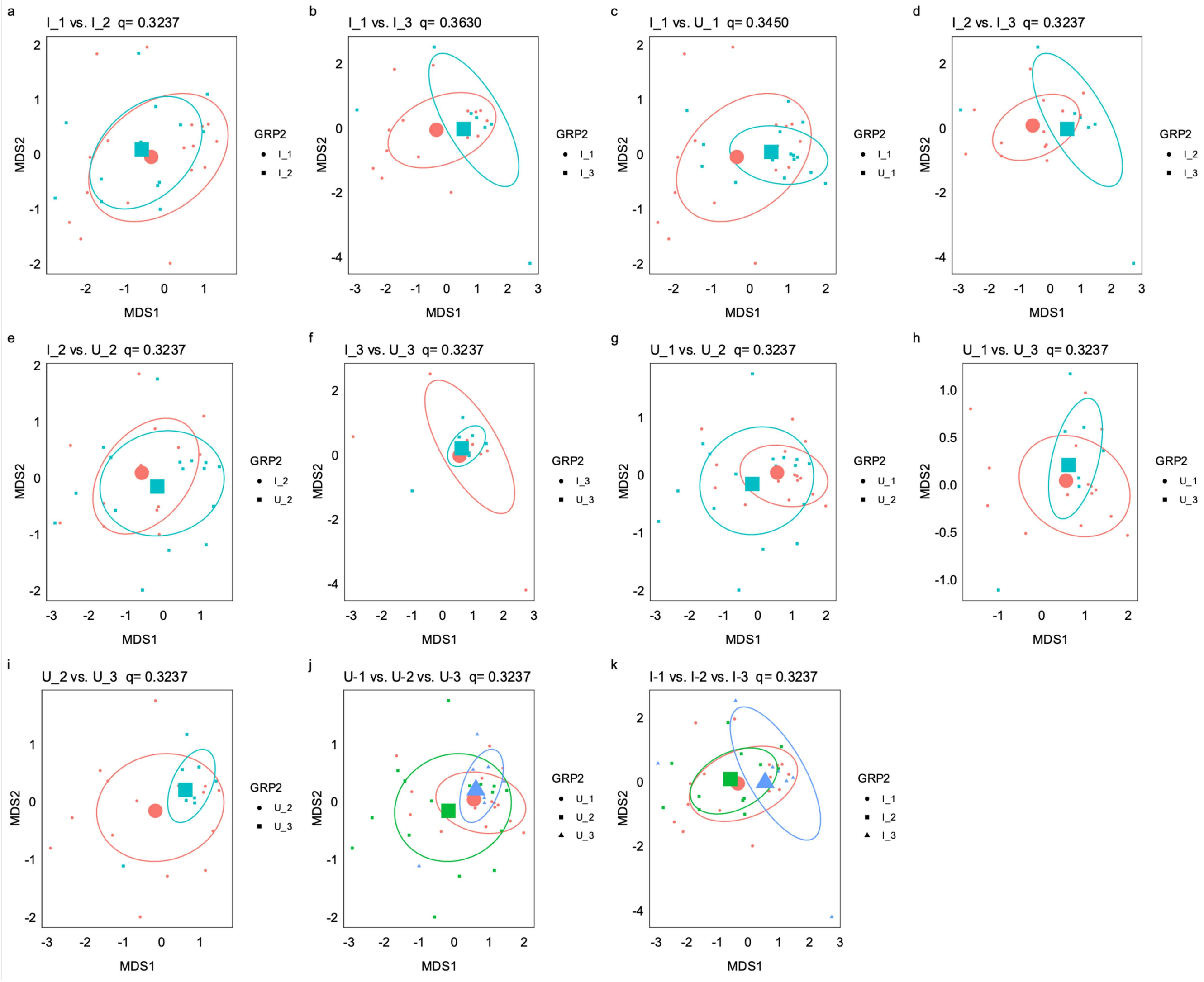
a
I_1 vs. I_2 q= 0.3237
b
I_1 vs. I_3 q= 0.3630
c
I_1 vs. U_1 q= 0.3450
d
I_2 vs. I_3 q= 0.3237
e
I_2 vs. U_2 q= 0.3237
f
I_3 vs. U_3 q= 0.3237
g
U_1 vs. U_2 q= 0.3237
h
U_1 vs. U_3 q= 0.3237
i
U_2 vs. U_3 q= 0.3237
j
U-1 vs. U-2 vs. U-3 q= 0.3237
k
I-1 vs. I-2 vs. I-3 q= 0.3237
MDS1
MDS2
GRP2

Figure S13: Pair and treatment group-wise comparisons of nMDS clustering for Oral AMR. Q values represent p values adjusted for multiple comparisons using the FDR correction.

Figure S14

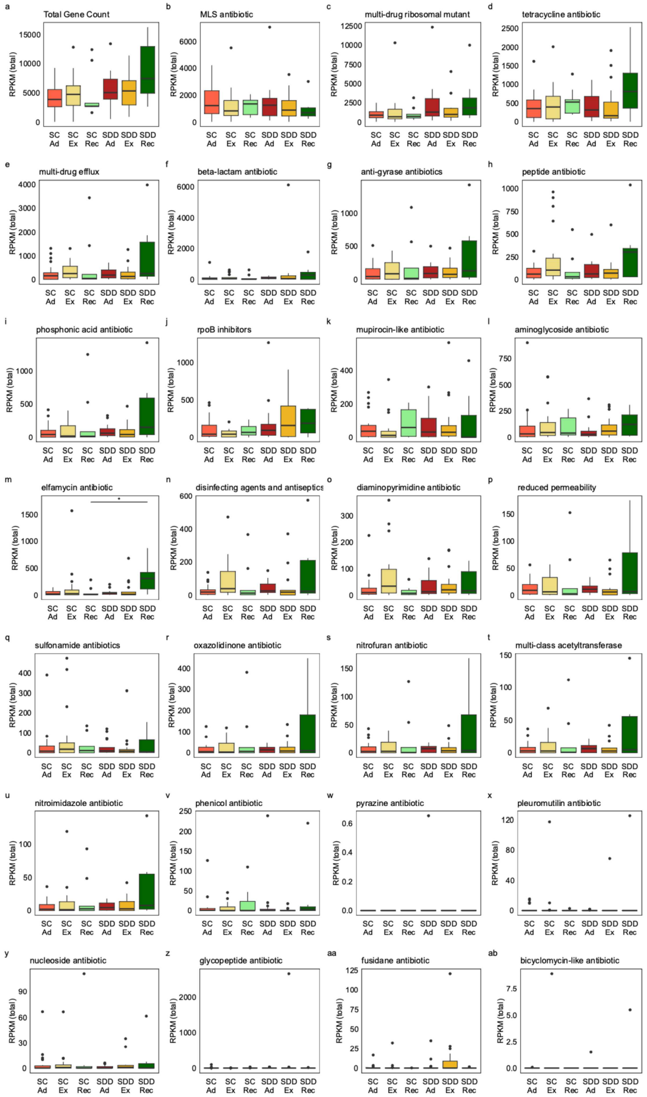
a Total Gene Count
b MLS antibiotic
c multi-drug ribosomal mutant
d tetracycline antibiotic
e multi-drug efflux
f beta-lactam antibiotic
g anti-gyrase antibiotics
h peptide antibiotic
i phosphonic acid antibiotic
j rpoB inhibitors
k mupirocin-like antibiotic
l aminoglycoside antibiotic
m elfamycin antibiotic
n disinfecting agents and antiseptics
o diaminopyrimidine antibiotic
p reduced permeability
q sulfonamide antibiotics
r oxazolidinone antibiotic
s nitrofuran antibiotic
t multi-class acetyltransferase
u nitroimidazole antibiotic
v phenicol antibiotic
w pyrazine antibiotic
x pleuromutilin antibiotic
y nucleoside antibiotic
z glycopeptide antibiotic
aa fusidane antibiotic
ab bicyclomycin-like antibiotic
RPKM (total)
SC Ad
SC Ex
SC Rec
SDD Ad
SDD Ex
SDD Rec

BMJ Publishing Group Limited (BMJ) disclaims all liability and responsibility arising from any reliance placed on this supplemental material which has been supplied by the author(s)

Figure S14: Antimicrobial resistance changes in the faecal microbiome of critically ill children. Antimicrobial resistance genes assembled using ARIBA against the CARD database v 3.2.7 were examined to determine differences between standard care and SDD-enhanced infection control. Gene counts were normalised as reads per kilobase(gene) per million sequencing reads (RPKM). A) Total gene count, b) Macrolides, Lincosamides, and Streptogramins (MLS), c) Multi-compound ribosomal mutants, d) Tetracyclines, e) Multi-drug efflux, f) beta-lactams, g) gyrase and topoisomerase inhibitors, h) peptide antibiotics (including polymyxin), i) phosphonic acid and derivatives, j) inhibitors of rpoB, k) mupirocin-like compounds, l) aminoglycosides, m) elfamycins, n) disinfecting agents and detergents (triclosan), o) diaminopyrimidines, p) sulfonamides, q) genes reducing permeability to antimicrobials, r) oxazolidinone compounds, s) nitrofurans, t) nitroimidazoles, u) multi-drug acetyltransferases, v) phenicols, w) pyrazines, x) pleuromutilins, y)nucleosides, z) glycopeptides, aa) fusidane, ab) bicyclomycin-like.

Figure S15

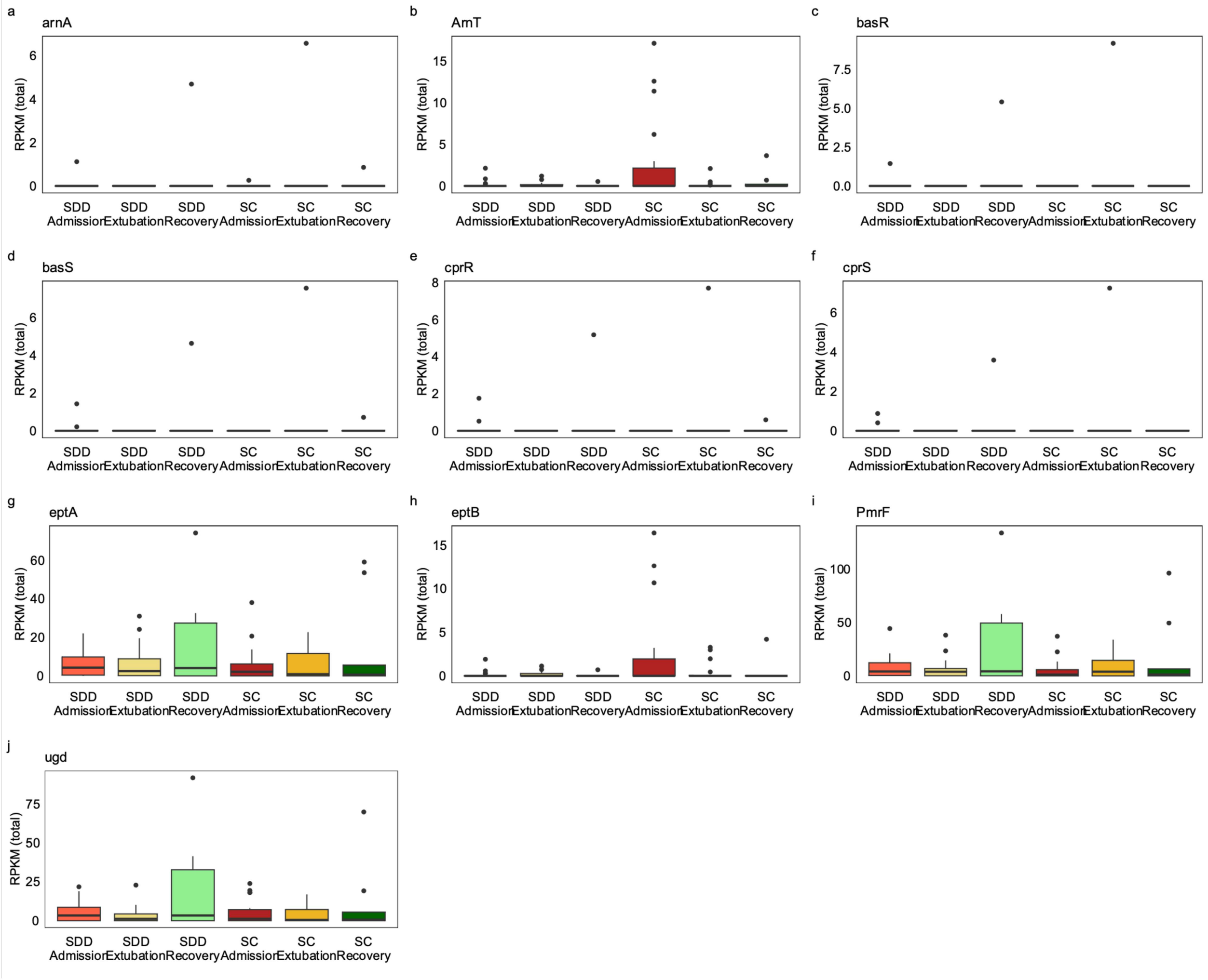
a
arnA
b
ArnT
c
basR
d
basS
e
cprR
f
cprS
g
eptA
h
eptB
i
PmrF
j
ugd
RPKM (total)
SDD Admission
SDD Extubation
SDD Recovery
SC Admission
SC Extubation
SC Recovery

Figure S15: Comparisons of Colistin resistance genes. Multiple comparisons performed with repeated Wilcoxon tests and p values adjusted using FDR.

Figure S16

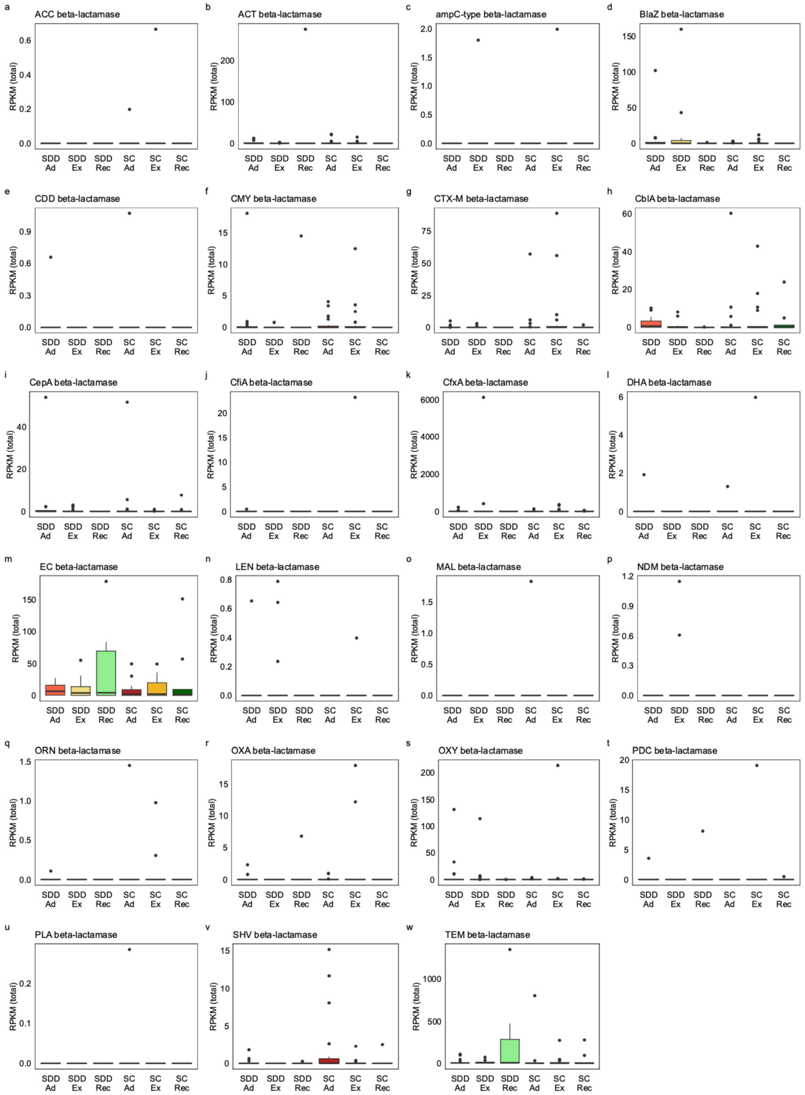
a ACC beta-lactamase
b ACT beta-lactamase
c ampC-type beta-lactamase
d BlaZ beta-lactamase
e CDD beta-lactamase
f CMY beta-lactamase
g CTX-M beta-lactamase
h CblA beta-lactamase
i CepA beta-lactamase
j CfiA beta-lactamase
k CfxA beta-lactamase
l DHA beta-lactamase
m EC beta-lactamase
n LEN beta-lactamase
o MAL beta-lactamase
p NDM beta-lactamase
q ORN beta-lactamase
r OXA beta-lactamase
s OXY beta-lactamase
t PDC beta-lactamase
u PLA beta-lactamase
v SHV beta-lactamase
w TEM beta-lactamase
RPKM (total)
SDD Ad
SDD Ex
SDD Rec
SC Ad
SC Ex
SC Rec

Figure S16: Comparisons of beta-lactamase genes. Multiple comparisons performed with repeated Wilcoxon tests and p values adjusted using FDR.

Figure S17

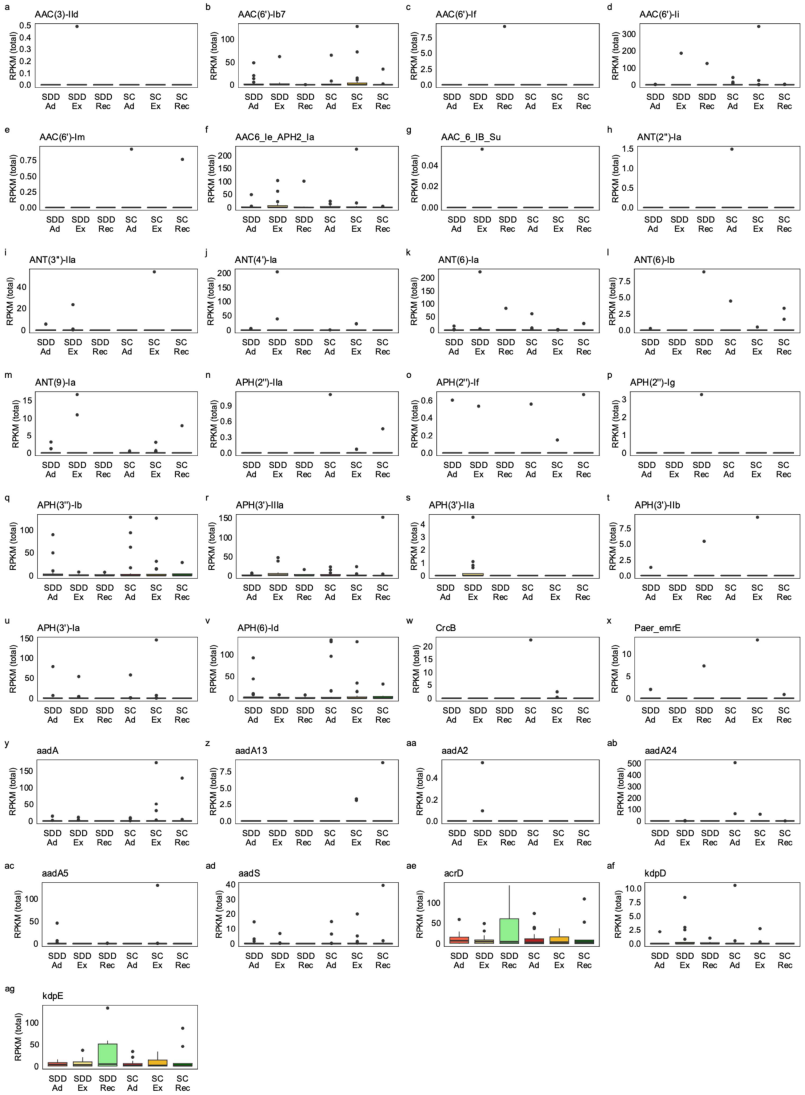
a AAC(3)-IId
b AAC(6')-Ib7
c AAC(6')-If
d AAC(6')-Ii
e AAC(6')-Im
f AAC6_Ie_APH2_Ia
g AAC_6_IB_Su
h ANT(2")-Ia
i ANT(3")-IIa
j ANT(4')-Ia
k ANT(6)-Ia
l ANT(6)-Ib
m ANT(9)-Ia
n APH(2")-IIa
o APH(2")-If
p APH(2")-Ig
q APH(3")-Ib
r APH(3')-IIIa
s APH(3')-IIa
t APH(3')-IIb
u APH(3')-Ia
v APH(6)-Id
w CrcB
x Paer_emrE
y aadA
z aadA13
aa aadA2
ab aadA24
ac aadA5
ad aadS
ae acrD
af kdpD
ag kdpE
RPKM (total)
SDD Ad
SDD Ex
SDD Rec
SC Ad
SC Ex
SC Rec

BMJ Publishing Group Limited (BMJ) disclaims all liability and responsibility arising from any reliance placed on this supplemental material which has been supplied by the author(s)

Figure S17: Comparisons of aminoglycoside resistance genes. Multiple comparisons performed with repeated Wilcoxon tests and p values adjusted using FDR. Genes **AAC(3)-IId, AAC(6')-Ib7, AAC(6')-If, AAC(6')-Ii, AAC(6')-Im**, **AAC(6)-Ie-APH(2)-Ia**, **AAC(6')-IB-Su, ANT(2")-Ia**, **APH(2")-IIa, APH(2")-If, APH(2")-Ig** are reported in the literature to confer tobramycin resistance.
